# BCMA‐Engineered Dendritic Cell‐Derived Exosomes as Bi‐Functional Therapeutics Orchestrating Cytokine Sequestration and Immune Activation for Multiple Myeloma

**DOI:** 10.1002/advs.75686

**Published:** 2026-05-15

**Authors:** Yuqing Zeng, Chao He, Zhibin He, Hongbo Chen, Fang Cheng, Yongjiang Zheng

**Affiliations:** ^1^ School of Pharmaceutical Sciences (Shenzhen) Sun Yat‐sen University Shenzhen China; ^2^ Department of Hematology Institute of Hematology The Third Affiliated Hospital of Sun Yat‐sen University Guangzhou China; ^3^ Department of Hematology Zhaoqing Hospital The Third Affiliated Hospital of Sun Yat‐sen University Zhaoqing China; ^4^ Department of Endocrinology Southwest Hospital Army Medical University (The Third Military Medical University) Chongqing China

**Keywords:** APRIL/BAFF axis, BCMA, cell‐free therapy, dendritic cell‐derived exosomes, multiple myeloma

## Abstract

The immunosuppressive bone marrow microenvironment (BMM) and cytokine dysregulation remain major barriers to curing multiple myeloma (MM). Despite the promise of B‐cell maturation antigen (BCMA)‐targeted therapies, their clinical utility is often limited by antigen escape and insufficient immune activation. Here, we developed DB Exo, a cell‐free therapeutic platform utilizing allogeneic dendritic cell‐derived exosomes engineered to surface display BCMA. Mechanistically, DB Exo act as molecular decoys that predominantly sequester soluble APRIL with partial BAFF attenuation, thereby effectively disrupting NF‐κB pro‐survival signaling in MM cells. Concurrently, DB Exo retain inherited costimulatory molecules (CD80, CD86, and MHC‐II) to trigger strong host immune activation, expanding CD8^+^ T cells and enhancing the secretion of cytotoxic effector molecules. In an orthotopic murine model, DB Exo suppress tumor burden by ∼72% and remodel the BMM by increasing cytotoxic T‐lymphocyte infiltration and elevating serum IFN‐γ and Granzyme B levels. The robust antitumor efficacy was further validated in a subcutaneous model, with DB Exo achieving a ∼75% reduction in tumor weight. Our findings establish DB Exo as a potent bi‐functional exosome platform that integrates targeted cytokine blockade with in situ immune activation, offering a promising cell‐free strategy for MM treatment.

## Graphical abstract

A dual‐function cell‐free therapeutic based on DC2.4 cell‐derived exosomes engineered to display BCMA. (**Left**) Soluble Ligand Sequestration (Decoy Function): DB Exo act as molecular decoys that predominantly sequester soluble APRIL with partial BAFF attenuation, effectively disrupting the NF‐κB survival signaling axis and suppressing myeloma cell proliferation. (**Right**) Host Immune Activation (Adjuvant‐like Function): Through inherited costimulatory molecules (CD80, CD86) and MHC‐II, DB Exo reverse the immunosuppressive bone marrow microenvironment by priming host CD8^+^ T cells to secrete cytotoxic effectors, including IFN‐γ, Granzyme B, and Perforin. Together, these effects lead to tumor regression in murine multiple myeloma models.

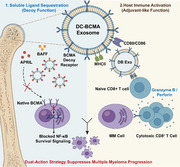

## 1 Introduction

Multiple myeloma (MM) is a hematological malignancy defined by the clonal expansion of malignant plasma cells within the bone marrow [1]. Over the past two decades, the treatment of MM has changed significantly following the clinical implementation of proteasome inhibitors (PIs), immunomodulatory drugs (IMiDs), and anti‐CD38 monoclonal antibodies [2, 3]. These interventions have extended the median overall survival of patients [4, 5]. Nevertheless, MM remains incurable for the vast majority, with a 5‐year relative survival rate of approximately 62% [6]. A significant clinical challenge is the emergence of multi‐drug resistance and the inevitable cycle of relapse, eventually leading to a treatment‐resistant state [7]. Emerging data in myeloma research suggest that this persistence is not merely a consequence of tumor‐intrinsic genetic instability but is critically supported by the specialized bone marrow microenvironment (BMM) [8, 9, 10].

The BMM is a complex, multi‐component system comprising cellular elements, including mesenchymal stem cells, osteoblasts, osteoclasts, and various immune subsets, as well as a non‐cellular compartment consisting of the extracellular matrix and a rich collection of soluble factors [11, 12]. Within this protective niche, malignant plasma cells are shielded from both cytotoxic drugs and endogenous immune recognition [11, 12]. Pro‐survival cues are delivered through direct physical interactions and the sustained availability of soluble cytokines, which collectively create a reservoir for minimal residual disease [12]. Consequently, therapeutic strategies must move beyond targeting the tumor cells in isolation and instead address the intricate signaling networks and immunosuppressive barriers established within the BMM.

A key mediator of this microenvironmental interaction is the B‐cell maturation antigen (BCMA), a member of the tumor necrosis factor receptor (TNFR) superfamily [13]. BCMA expression is highly selective, restricted almost exclusively to late‐stage B cells and plasma cells, making it an effective therapeutic target [13]. The biological role of BCMA in MM is linked to its interaction with two primary ligands: a proliferation‐inducing ligand (APRIL) and B‐cell activating factor (BAFF) [14]. These ligands are predominantly secreted by myeloid cells, such as osteoclasts and macrophages, within the BMM [14]. Upon binding, BCMA activates a signaling cascade involving the non‐canonical NF‐κB, MAPK, and PI3K/Akt pathways [14, 15]. These intracellular events are essential for plasma cell homeostasis, driving proliferation and upregulating anti‐apoptotic proteins such as Mcl‐1 and Bcl‐2 [14]. Persistent activation of the APRIL/BAFF–BCMA axis promotes tumor growth and contributes to the failure of conventional therapies, demonstrating that this pathway is a critical factor in MM pathobiology [14].

The success of BCMA‐directed immunotherapies, particularly cell‐based approaches like chimeric antigen receptor (CAR) T‐cell therapies and bispecific T‐cell engagers (BiTEs), has confirmed BCMA as an important therapeutic target [16, 17, 18]. However, despite high initial response rates in relapsed/refractory MM, long‐term disease control remains limited [16, 17]. Several resistance mechanisms have been identified, including the downregulation of surface BCMA or gamma‐secretase‐mediated shedding, which produces soluble BCMA (sBCMA) that acts as a decoy [19, 20]. Crucially, while CAR‐T cells act as potent immune effectors, their function is often severely compromised by the immunosuppressive BMM, leading to rapid T‐cell exhaustion [21, 22]. Additionally, attempts to target individual ligands, such as BAFF‐neutralizing antibodies, have shown modest results, likely due to the redundant signaling provided by APRIL [23, 24, 25]. These observations highlight the need for an approach that can concurrently neutralize multiple survival ligands while overcoming microenvironmental suppression. In our previous work, we validated the feasibility of this ligand‐scavenging strategy by engineering nanovesicles derived from HEK‐293T cells to reconstruct the BCMA receptor (Re‐BCMA‐NVs) [26]. We demonstrated that these vesicles could effectively act as “molecular decoys,” sequestering soluble APRIL and BAFF, blocking NF‐κB signaling, and sensitizing MM cells to bortezomib treatment. However, as they were derived from non‐immune kidney cells, these vesicles functioned solely as a passive blockade and lacked the intrinsic ability to reactivate host immunity. Consequently, to achieve durable disease control, there is a critical need to further develop this platform into one that can simultaneously deprive tumor cells of survival ligands and restore the antitumor immune response.

Dendritic cell (DC)‐based vaccines have been studied as a method to re‐establish antitumor immunity in MM [27]. DCs are potent professional antigen‐presenting cells (APCs), capable of bridging innate and adaptive immunity by priming cytotoxic T lymphocytes (CTLs) [27]. However, clinical trials using autologous DC vaccines in MM patients have frequently failed to meet expectations [28]. This failure is largely attributed to DC dysfunction induced by the tumor. Myeloma‐derived factors, such as interleukin‐6 (IL‐6) and vascular endothelial growth factor (VEGF), impair the maturation and antigen‐presenting capacity of endogenous DCs, making them unable to mount an effective immune response [29, 30]. To bypass the limitations of cell‐based therapies, dendritic cell‐derived exosomes (DC Exo) have emerged as a cell‐free alternative. DC Exo are nano‐sized extracellular vesicles that inherit the immunological machinery of their parental cells, including MHC class I and II complexes and essential costimulatory molecules like CD80 and CD86 [31]. Unlike live DCs, exosomes are resistant to tumor‐mediated immunosuppression and show enhanced stability and bioavailability [32, 33]. Furthermore, DC Exo are enriched in adhesion molecules such as ICAM‐1, which facilitates their interaction with recipient immune cells and supports efficient antigen transfer and T‐cell priming [34]. These properties position DC Exo as a stable, cell‐free platform for delivering immunostimulatory signals and enhancing antitumor immune responses.

To address these challenges, we developed DB Exo, an allogeneic DC‐derived exosome platform engineered to surface display BCMA. Our findings demonstrate that DB Exo effectively access the bone marrow niche, where they neutralize soluble APRIL/BAFF and disrupt ligand‐mediated survival signaling. Concurrently, the surface‐displayed costimulatory molecules provide the necessary signals to activate the endogenous T‐cell population, leading to significant suppression of both orthotopic and ectopic myeloma growth. These results suggest that decoy‐receptor‐functionalized DC exosomes represent a robust cell‐free strategy to overcome microenvironment‐mediated resistance in multiple myeloma.

## 2 Results

### 2.1 Engineering and Characterization of BCMA‐Displaying DC‐Derived Exosomes

**FIGURE 1:**
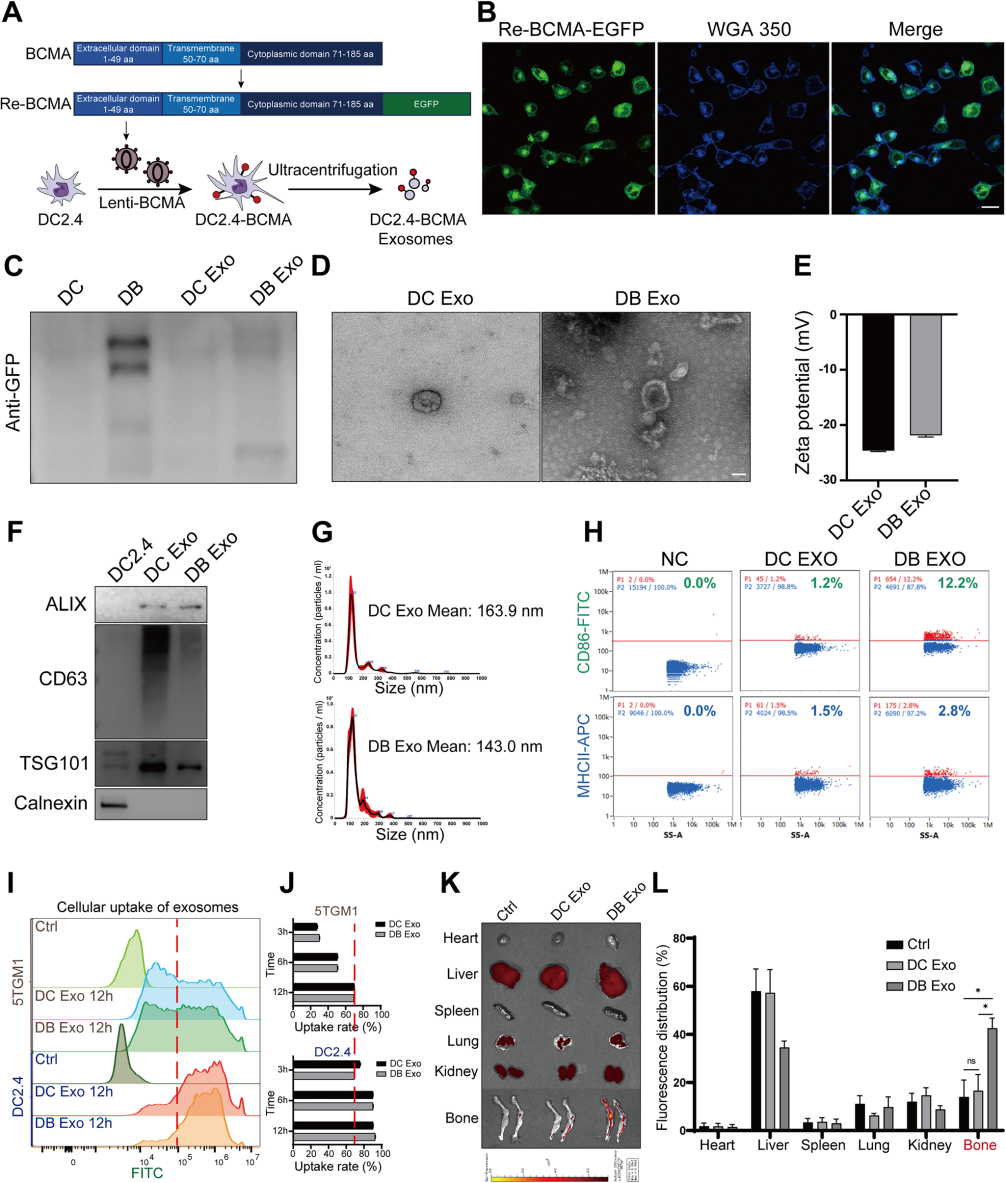
**Engineering and characterization of BCMA‐displaying dendritic cell exosomes (DB Exo)**. (A) Schematic illustration of the lentiviral construct encoding the BCMA‐EGFP fusion protein and the workflow for isolating engineered exosomes from stably transduced DC2.4 cells. (B) Confocal laser scanning microscopy (CLSM) images of receptor localization. DC2.4 cells expressing the BCMA‐EGFP fusion protein (green) were counterstained with the membrane marker WGA (blue). Scale bar: 10 µm. (C) Western blot analysis of BCMA‐GFP fusion protein expression in cell lysates and purified exosomes from mock‐transduced (DC) and BCMA‐overexpressing (DB) cells. (D) Transmission electron microscopy (TEM) images showing the morphology of DC Exo and DB Exo. Scale bar: 100 nm. (E) Zeta potential measurements of DC Exo and DB Exo. *n =* 3. (F) Western blot analysis of exosomal marker proteins (ALIX, CD63, TSG101) and the endoplasmic reticulum‐resident protein Calnexin in cell lysates (DC2.4) and purified exosomes (DC Exo and DB Exo). (G) Nanoparticle tracking analysis (NTA) profiles showing particle size distribution and concentration. (H) Nano‐flow cytometry dot plots showing single‐particle surface expression of CD86‐FITC (upper panels) and MHC‐II‐APC (lower panels) on DC Exo and DB Exo compared to unstained negative control (NC). The percentage of marker‐positive particles (P1, red) is indicated in each plot. (I) Flow cytometry analysis and (J) quantification of exosome uptake kinetics in DC2.4 and 5TGM1 cells over a 12‐h period. *n =* 3. (K) Ex vivo fluorescence imaging of major organs and femurs excised 12 h after intravenous injection of Cy5‐labeled exosomes, demonstrating biodistribution and bone marrow accumulation. (L) Quantitative analysis of organ‐specific fluorescence distribution, expressed as the percentage of total fluorescence signal across all harvested organs, for Ctrl, DC Exo, and DB Exo groups at 12 h post‐injection. DB Exo exhibited significantly greater bone accumulation compared to DC Exo. *n* = 3. Data in panel **L** are shown as mean ± SD. Statistical significance was analyzed using one‐way ANOVA followed by Tukey's multiple comparisons test. ^*^ *p* < 0.05, ns: not significant.

To simultaneously target BMM cytokine dysregulation and the immunosuppressive microenvironment in MM, the murine dendritic cell line DC2.4 was genetically modified to express a BCMA decoy receptor. A lentiviral vector encoding a fusion protein consisting of full‐length mouse BCMA and an EGFP tag was stably transduced into DC2.4 cells to establish the stable DB cell line (Figure 1A). Confocal microscopy confirmed the precise membrane localization of the fusion protein, as evidenced by the substantial colocalization of the EGFP signal with the plasma membrane marker wheat germ agglutinin (WGA) (Figure 1B). Subsequent immunoblotting using anti‐GFP antibodies detected a specific band corresponding to the BCMA‐EGFP fusion protein in both the engineered DB cells and the secreted DB exosomes (DB Exo), confirming the successful sorting of the therapeutic cargo into the vesicles (Figure 1C).

We next assessed whether the genetic engineering affected the physicochemical properties of the exosomes. Transmission electron microscopy revealed that DB Exo retained the classic cup‐shaped morphology typical of exosomes, indistinguishable from unmodified DC exosomes (Figure 1D). Zeta potential analysis indicated that both groups maintained a comparable negative surface charge (approximately −22 mV), suggesting that the surface display of BCMA did not compromise the colloidal stability of the vesicles (Figure 1E). Western blot analysis further confirmed the exosomal identity, showing distinct enrichment of the hallmark proteins ALIX, CD63, and TSG101 in the vesicle fractions compared to cell lysates (Figure 1F). Nanoparticle tracking analysis (NTA) showed that DB Exo maintained a size distribution (mean diameter 143 nm) comparable to that of control exosomes, suggesting that the surface display of BCMA did not induce aggregation or aberrant vesicle formation (Figure 1G).

To characterize the retention of surface antigen‐presenting markers, nano‐flow cytometry was employed to profile CD86 and MHC‐II expression at the single‐particle level (Figure 1H). DB Exo exhibited a CD86 positivity rate of 12.2% (compared to 1.2% for DC Exo) and an MHC‐II positivity rate of 2.8% (compared to 1.5% for DC Exo). As these values remained well above the NC baseline, these results confirm that DB Exo preserve the costimulatory phenotype essential for mediating T‐cell immune activation.

The cellular uptake of DB Exo by target cell populations was then evaluated. Confocal imaging of bone marrow‐derived dendritic cells (BMDCs) incubated with Cy5‐labeled exosomes revealed robust intracellular accumulation within 6 h, confirming efficient internalization by primary antigen‐presenting cells (Figure S1A). Kinetic analysis via flow cytometry demonstrated that DC2.4 cells internalized the exosomes rapidly, with uptake rates exceeding 90% at 12 h, whereas 5TGM1 myeloma cells exhibited a slower uptake profile (∼60%) (Figure 1I,J). This efficient uptake by dendritic cells supports the potential of DB Exo to function as an immunomodulatory vesicle platform capable of engaging antigen‐presenting cells. Finally, in vivo biodistribution studies showed that following intravenous injection, liver accumulation was predominant across all groups, consistent with reticuloendothelial clearance (Figure 1K). Quantitative analysis of organ‐specific fluorescence distribution revealed that DB Exo achieved significantly greater bone accumulation than DC Exo (*p* < 0.05), whereas no significant difference was observed in other organs (Figure 1L; Figure S1B). This enhanced bone accumulation suggests that DB Exo can effectively access the myeloma niche, supporting the platform's dual therapeutic potential.

### 2.2 DB Exo Function as Molecular Decoys to Suppress MM Survival Signaling

**FIGURE 2:**
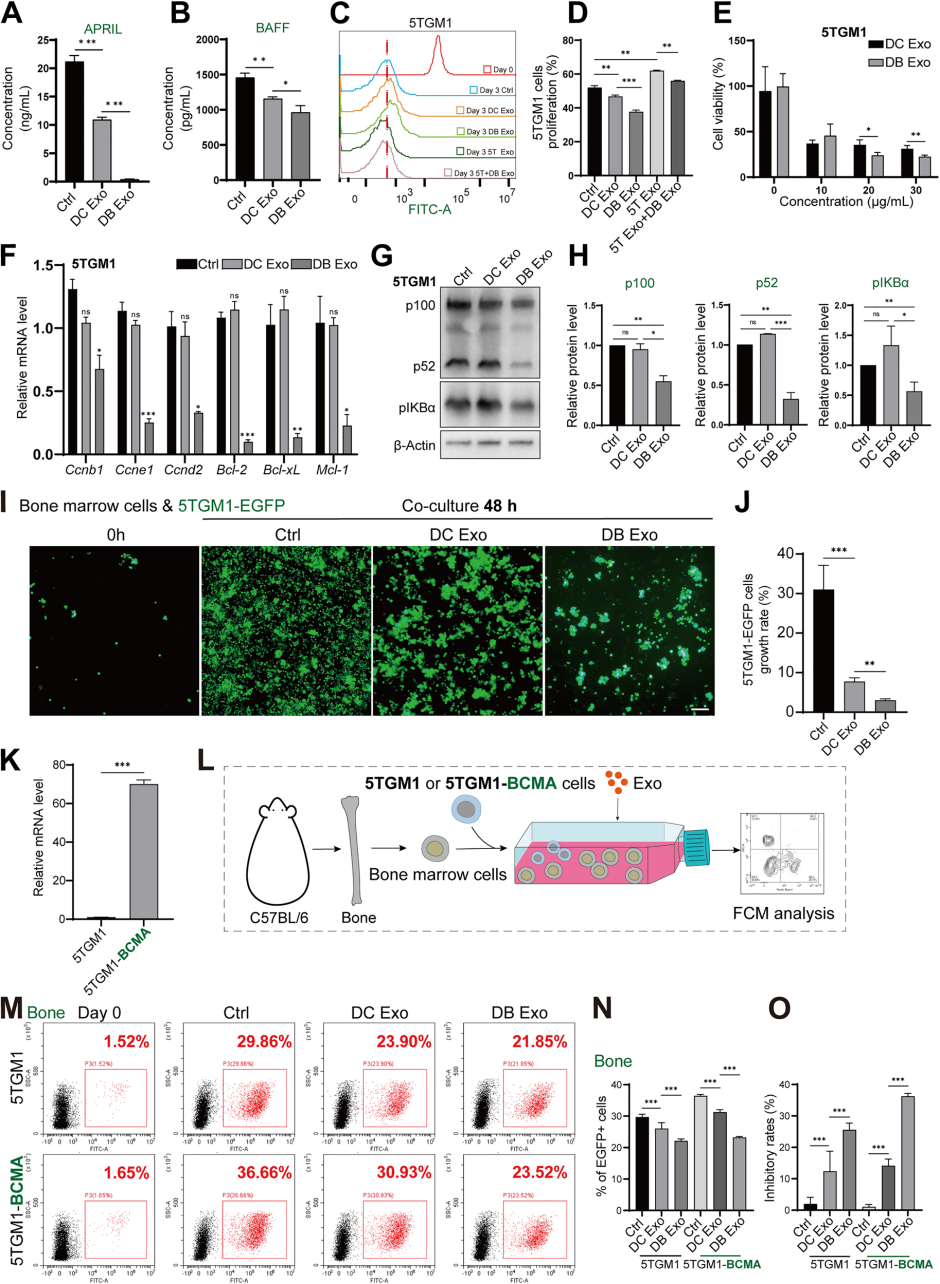
**DB Exo impair multiple myeloma survival signaling via ligand sequestration**. (A, B) ELISA quantification of soluble APRIL (A) and BAFF (B) levels in culture supernatants following incubation with PBS (Ctrl), DC Exo, or DB Exo. *n =* 3. (C) Representative flow cytometry histograms and (D) quantification of 5TGM1 cell proliferation assessed by CFSE proliferation assay. Cells were cultured with the indicated exosomes, including tumor‐derived exosomes (5T Exo), for 72 h. *n =* 3. (E) Cell viability of 5TGM1 cells treated with varying concentrations of exosomes for 48 h, determined by CCK‐8 assay. *n =* 6. (F) RT‐qPCR analysis of cell cycle and apoptosis‐related gene expression (*Ccnb1*, *Ccne1*, *Ccnd2*, *Bcl‐2*, *Bcl‐xL*, *Mcl‐1*) in 5TGM1 cells post‐treatment. Relative mRNA levels were calculated using the 2^−ΔΔCt^ method and normalized to the Ctrl group. *n =* 3. (G) Western blot analysis of NF‐κB signaling pathways. Markers for both the non‐canonical (processing of p100 to p52) and canonical (pIκBα) were assessed in 5TGM1 cells. (H) Densitometric quantification of the protein bands in (G), normalized to β‐Actin and expressed relative to the Ctrl group. *n =* 3. (I) Representative fluorescence microscopy images of 5TGM1‐EGFP cell growth in an ex vivo bone marrow co‐culture system after 48 h of treatment. Scale bar: 100 µm. (J) Quantification of the tumor cell growth rate based on GFP^+^ signals. The growth rate was calculated using the following formula: Growth Rate (%) = [(Mean particle count at 48 h—Mean particle count at 0 h) / Mean particle count at 0 h] × 100%. *n =* 3. (K) Relative mRNA expression of BCMA in wild‐type 5TGM1 and engineered 5TGM1‐BCMA cell lines. *n =* 3. (L) Schematic illustration of the competitive ex vivo co‐culture assay. (M) Flow cytometry plots and (N) quantification of the percentage of residual EGFP^+^ tumor cells (wild‐type vs. BCMA‐overexpressing) in the bone marrow co‐culture system. *n =* 3. (O) Calculated inhibitory rates comparing the sensitivity of 5TGM1 and 5TGM1‐BCMA cells to DB Exo treatment. The inhibitory rate was calculated as follows: Inhibitory Rate (%) = [(Mean % in Control—Individual % in Treatment) / Mean % in Control] × 100%. Statistical significance was analyzed using one‐way ANOVA followed by Tukey's multiple comparisons test for panels A, B, D, F, H, J, N, and O. Unpaired two‐tailed Student's t‐test was used for panels E and K. All data are presented as mean ± SD. ^*^ *p* < 0.05, ^**^ *p* < 0.01, ^***^ *p* < 0.001, ns: not significant.

With the structural integrity and improved bone marrow niche access established, the functional capacity of DB Exo to disrupt cytokine‐dependent survival signaling within the MM niche was next evaluated. To validate their role as molecular decoys, we incubated the exosomes with supernatants containing myeloma‐associated survival factors. ELISA quantification revealed that DB Exo acted as potent ligand‐sequestering decoys, resulting in near‐complete depletion of soluble APRIL (reducing levels from approximately 21 pg/mL to undetectable limits, >98% sequestration) and a significant reduction in BAFF (∼35%) (Figure 2A,B). The results demonstrate that the surface‐displayed BCMA decoy receptor effectively sequesters APRIL, with partial BAFF attenuation.

To dissect the respective contributions of APRIL and BAFF to myeloma proliferation, concentration‐dependent CCK‐8 assays were performed for each ligand individually (Figure S1C,D). APRIL stimulation produced a sustained, dose‐dependent increase in 5TGM1 proliferation across a broad concentration range (31.25–500 ng/mL), with the effect plateauing at higher doses, suggesting receptor saturation. In contrast, BAFF exhibited a biphasic response: proliferative enhancement was observed at low concentrations (31.25–62.5 ng/mL), whereas progressive inhibition emerged above 500 ng/mL. Based on these dose‐response profiles, an optimal proliferative concentration of approximately 50 ng/mL was selected for each ligand in subsequent assays. Under these conditions, APRIL alone markedly increased proliferation (OD from ∼0.9 to ∼2.2, *p* < 0.001), whereas BAFF alone produced only a modest, nonsignificant increase (OD ∼1.1), establishing APRIL as the dominant mitogenic driver (Figure S1E). DB Exo effectively suppressed APRIL‐driven proliferation to near‐baseline levels (*p*<0.01), while exerting no significant effect on BAFF‐stimulated cells. Under combined APRIL+BAFF stimulation, which yielded the highest proliferation (∼2.7), DB Exo reduced proliferation to ∼1.5 (*p*<0.001), confirming that the therapeutic effect is primarily attributable to APRIL neutralization.

At the signaling level, Western blot analysis further corroborated these findings (Figure S1F). BAFF stimulation alone induced marginal elevation of p52 (1.2) and pIκBα (1.5), and DB Exo addition produced no appreciable change in either marker (p52: 1.3; pIκBα: 1.4), consistent with the limited BAFF clearance (∼35%) and the weak intrinsic signaling potency of BAFF. APRIL stimulation alone substantially activated both NF‐κB pathways, elevating p52 to 1.6 and pIκBα to 2.2. DB Exo treatment in APRIL‐stimulated cells reduced p52 to 0.9 and pIκBα to 0.6, demonstrating that near‐complete APRIL sequestration effectively suppresses both canonical and non‐canonical NF‐κB signaling. Together, these results establish that APRIL is the dominant survival ligand in this axis, and that DB Exo exert their primary therapeutic effect through near‐complete APRIL sequestration, with BAFF clearance playing a limited supplementary role.

Having established the ligand‐specific contributions at the molecular level, the overall physiological impact of DB Exo‐mediated ligand deprivation on tumor proliferation was further quantified via CFSE proliferation assay (Figure 2C,D). While tumor‐derived exosomes (5T Exo) typically accelerated malignant expansion, as indicated by an increase in the proliferation rate to 62.1%, DB Exo not only neutralized this stimulatory effect but also actively suppressed basal proliferation rates to 38.3%, significantly outperforming unmodified DC exosomes. This growth inhibition was confirmed to be dose‐dependent, establishing a direct correlation between decoy concentration and therapeutic efficacy (Figure 2E).

Mechanistically, we probed the downstream impact of ligand sequestration on the transcriptional and signaling landscape of the treated tumor cells. Quantitative PCR analysis revealed a broad downregulation of cell cycle drivers such as *Ccnb1*, *Ccne1*, and *Ccnd2* and key anti‐apoptotic regulators including *Bcl‐2*, *Bcl‐xL*, and *Mcl‐1* (Figure 2F). Western blot analysis linked these transcriptional changes to the suppression of NF‐κB signaling, a pivotal pathway in MM pathogenesis. The data revealed that DB Exo blocked the proteolytic processing of the non‐canonical p100 precursor into the active p52 subunit and reduced the phosphorylation of IκBα, a marker of the canonical pathway (Figure 2G,H). This dual inhibition suggests that DB Exo‐mediated ligand withdrawal effectively shuts down the constitutive NF‐κB activity required for myeloma survival.

To recapitulate the complexity of the bone marrow microenvironment, we utilized an ex vivo co‐culture system. Fluorescence imaging revealed that DB Exo treatment disrupted the formation of dense 5TGM1 tumor clusters, resulting in a sparse cellular distribution and a significant reduction in tumor cell growth rate compared to controls (Figure 2I,J). Finally, we addressed whether the therapeutic effect was dependent on the target cells' reliance on the BCMA axis. We engineered 5TGM1 cells to overexpress BCMA (Figure 2K), and confirmed that BCMA overexpression did not significantly alter the baseline growth kinetics of 5TGM1 cells (Figure S1G), ensuring that subsequent sensitivity to DB Exo‐mediated cytokine sequestration reflects signaling dependency rather than intrinsic growth differences. These cells were then subjected to a competitive inhibition assay (Figure 2L). Flow cytometric analysis demonstrated that while DB Exo inhibited wild‐type 5TGM1 growth by approximately 25%, the inhibitory rate reached approximately 35% in BCMA‐overexpressing cells (Figure 2M–O). Collectively, these differential sensitivities highlight a critical survival signaling dependency phenomenon: cells with higher BCMA expression possess a heightened dependency on APRIL/BAFF signaling for survival. Therefore, by starving the microenvironment of these ligands, DB Exo exert a differential growth‐inhibitory effect that preferentially suppresses aggressive, high‐antigen‐density malignant clones.

### 2.3 DB Exo Induce Dendritic Cell Maturation and Enhance Cytotoxic T Cell Immunity

**FIGURE 3:**
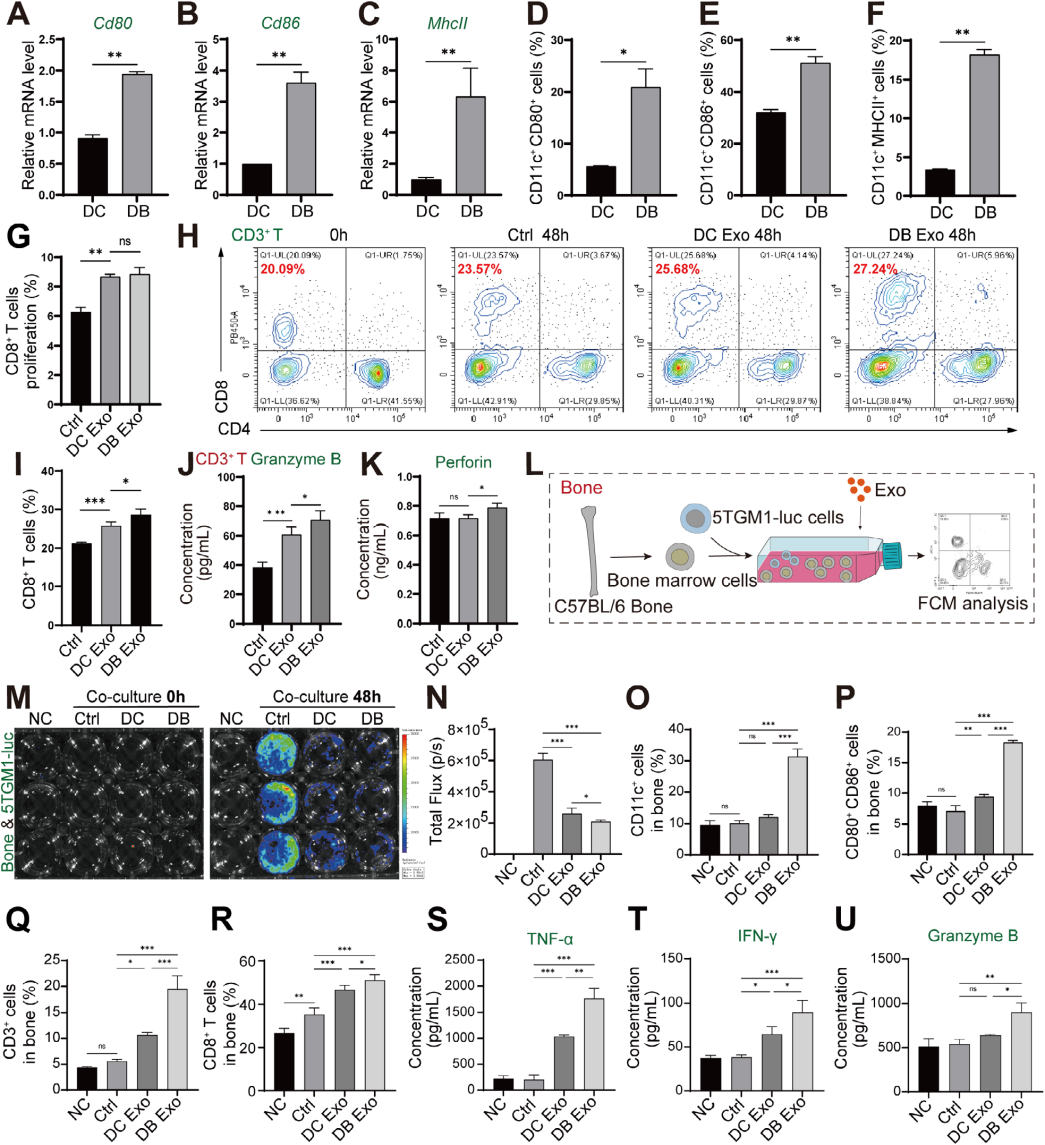
**DB Exo promote dendritic cell maturation and coordinate cytotoxic T cell responses ex vivo**. (A–C) Relative mRNA expression levels of *Cd80* (A), *Cd86* (B), and *MhcII* (C) in parental DC and DB cells assessed by RT‐qPCR. Relative mRNA levels were calculated using the 2^−ΔΔCt^ method and normalized to the DC group. *n =* 3. (D‐F) Flow cytometric quantification of the frequencies of CD11c^+^CD80^+^ (D), CD11c^+^CD86^+^ (E), and CD11c^+^MHC‐II^+^ (F) populations. *n =* 3. (G) Quantification of CD8^+^ T cell proliferation rates determined by CFSE dilution assay. *n =* 3. (H) Representative flow cytometry plots of CD3^+^ T cells illustrating the expansion of CD8^+^ T cell populations in a splenocyte co‐culture system following 48 h of treatment with PBS, DC Exo, or DB Exo. (I) Quantification of CD8^+^ T cell frequency from (H). *n =* 3. (J,K) ELISA measurement of Granzyme B (J) and Perforin (K) concentrations in co‐culture supernatants. *n =* 3. (L) Schematic diagram illustrating the ex vivo co‐culture system comprising C57BL/6 bone marrow cells and 5TGM1‐Luc cells to mimic the myeloma niche. (M,N) Bioluminescence imaging (M) and quantification of total bioluminescence flux (p/s) (N) assessing tumor burden in bone marrow co‐cultures after 48 h of treatment with PBS, DC Exo, or DB Exo. *n =* 3. (O,P) Flow cytometric analysis of the bone marrow myeloid compartment, quantifying the frequency of total CD11c^+^ dendritic cells (O) and mature CD80^+^CD86^+^ dendritic cells (P). *n =* 3. (Q,R) Flow cytometric analysis of the lymphoid compartment, showing the expansion of CD3^+^ T cells (Q) and cytotoxic CD8^+^ T cells (R) in the bone marrow. *n =* 3. (S–U) ELISA quantification of TNF‐α (S), IFN‐γ (T), and Granzyme B (U) concentrations in the co‐culture supernatants. *n =* 3. Unpaired two‐tailed Student's t‐test was used for panels A, B, C, D, E, and F. Statistical significance was analyzed using one‐way ANOVA followed by Tukey's multiple comparisons test for panels G, I, J, K, N, O, P, Q, R, S, T, and U. All data are presented as mean ± SD. ^*^ *p* < 0.05, ^**^ *p* < 0.01, ^***^ *p* < 0.001, ns: not significant.

Beyond direct ligand sequestration, we next investigated whether DB Exo could bridge innate and adaptive immunity. We first examined whether the engineering process altered the immunogenic phenotype of the parental DC2.4 cells, which would determine the inherited features of their secreted exosomes. Quantitative PCR analysis revealed a robust upregulation of key costimulatory and antigen‐presenting markers in DB cells compared to wild‐type DCs. Specifically, *Cd80*, *Cd86*, and *MhcII* mRNA levels were elevated by approximately 2.0‐, 3.5‐, and 6.0‐fold, respectively (Figure 3A–C). This transcriptional signature translated into a stable surface phenotype. The DB cells exhibited a significantly higher frequency of CD11c^+^ populations co‐expressing high densities of CD80, CD86, and MHC‐II (Figure 3D–F). These data indicate that BCMA engineering promoted the acquisition of a more mature antigen‐presenting phenotype in DC2.4 cells.

To determine whether this immunogenicity could be transferred via exosomes to elicit adaptive immunity, we employed a co‐culture system with murine splenic CD3^+^ T cells. Flow cytometric profiling performed after 48 h revealed that exposure to DB Exo induced a specific modulation of the T cell compartment. While overall CFSE‐based proliferation of CD8^+^ T cells was comparable between DB Exo and DC Exo groups (Figure 3G), DB Exo treatment enriched the cytotoxic CD8^+^ subset to 27.2% (Figure 3H,I) and promoted robust secretion of cytotoxic effector molecules, including Granzyme B (∼70 pg/mL) and Perforin (∼0.8 ng/mL) (Figure 3J,K). These findings demonstrate that DB Exo drive T cells into a functionally active cytotoxic state rather than merely inducing non‐specific expansion.

We next evaluated the therapeutic efficacy of DB Exo within the bone marrow microenvironment, the primary niche of MM progression and immunosuppression. An ex vivo bone marrow co‐culture system containing 5TGM1‐Luc cells was established (Figure 3L), and bioluminescence imaging demonstrated that DB Exo exerted marked tumor control, reducing total photon flux by approximately 3‐fold compared to controls (Figure 3M,N). Immunophenotyping revealed that DB Exo effectively remodeled the local immune landscape. Treatment triggered the recruitment and maturation of dendritic cells, increasing the CD11c^+^ population from 10.7% to 33.1% (Figure 3O; Figure S2A), and elevating the frequency of mature CD80^+^CD86^+^ DCs from 6.3% to 18.4% (Figure 3P; Figure S2B). Correspondingly, this restored antigen presentation promoted an enhanced T cell response within the bone marrow niche. The total CD3^+^ T cell population expanded (Figure 3Q; Figure S2C), and the proportion of CD8^+^ T cells increased to approximately 50% within the T cell compartment (Figure 3R; Figure S2D). This cellular remodeling was accompanied by a pro‐inflammatory cytokine profile, characterized by significantly elevated levels of TNF‐α (seven‐fold), IFN‐γ (five‐fold), and Granzyme B (five‐fold) in the bone marrow supernatants (Figure 3S–U). These results demonstrate that DB Exo can effectively overcome the local immunosuppressive conditions characteristic of the bone marrow niche to re‐establish antitumor immunity.

Finally, to assess the systemic reach of this immune modulation, we examined the splenic compartment using a splenocyte co‐culture system (Figure S3A). Consistent with the BM results, DB Exo treatment significantly suppressed tumor burden (Figure S3B,C) and enhanced peripheral immune activation. DB Exo increased the total frequency of mature DCs (Figure S3D–G) and induced a subsequent expansion of cytotoxic CD8^+^ T cells (Figure S3H–K). Functional verification confirmed that these effector cells were active, secreting high levels of the type 1 effector cytokines TNF‐α and IFN‐γ, as well as the cytotoxic molecule Granzyme B (Figure S3L–N). These findings indicate that DB Exo can induce DC maturation and promote the expansion of functional cytotoxic T cells within the splenic compartment.

Collectively, these findings demonstrate that DB Exo function as a dual‐mechanism therapeutic. They physically sequester soluble survival ligands while simultaneously overcoming local and systemic immunosuppression by promoting DC maturation and generating functional cytotoxic T cell responses.

### 2.4 DB Exo Show Potent Anti‐Myeloma Efficacy in an Orthotopic Model

**FIGURE 4:**
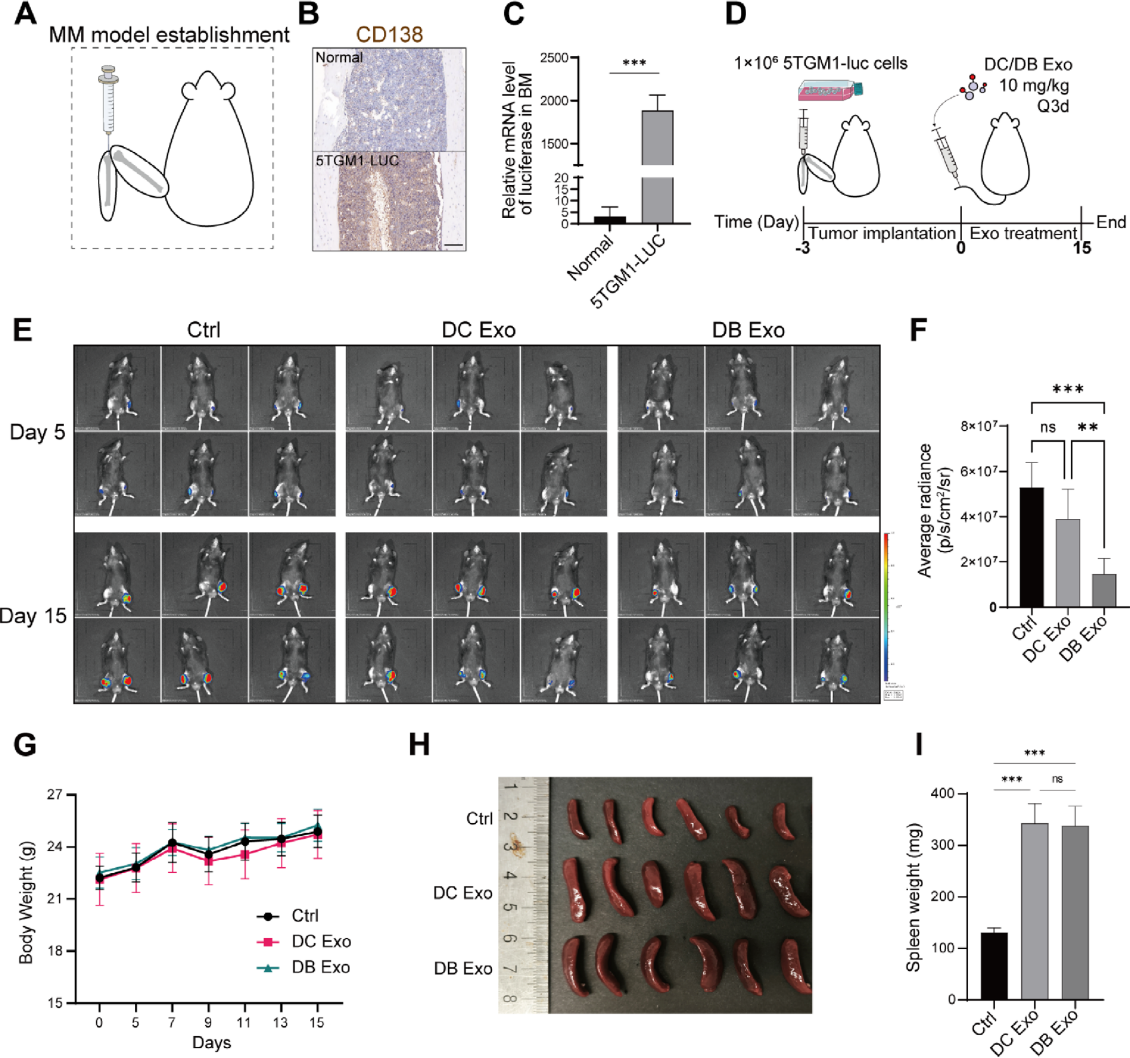
**DB Exo suppress multiple myeloma progression in an orthotopic immunocompetent mouse model**. (A) Schematic illustration of the orthotopic 5TGM1‐Luc multiple myeloma model establishment. (B) Representative immunohistochemical staining of CD138 in femoral bone marrow sections from normal and 5TGM1‐Luc tumor‐bearing mice. Scale bar: 50 µm. (C) Relative mRNA expression of luciferase in bone marrow tissue. *n =* 3. (D) Experimental timeline for tumor implantation and exosome treatment administration (10 mg/kg, i.v., every 3 days). (E) Longitudinal bioluminescence imaging of tumor burden in mice treated with PBS, DC Exo, or DB Exo on days 5 and 15 post‐treatment initiation. (F) Quantification of average bioluminescence radiance (p/s/cm^2^/sr) on day 15. *n =* 6. (G) Body weight monitoring curves of mice during the treatment period. (H,I) Macroscopic images (H) and spleen weight measurements (I) at the study endpoint. Unpaired two‐tailed Student's t‐test was used for panel C. Statistical significance was analyzed using one‐way ANOVA followed by Tukey's multiple comparisons test for panels F and I. All data are presented as mean ± SD. ^**^ *p* < 0.01, ^***^ *p* < 0.001, ns: not significant.

To evaluate the therapeutic efficacy of DB Exo within the pathological bone marrow microenvironment, a clinically relevant orthotopic model was established by the intrafemoral injection of luciferase‐labeled 5TGM1 cells into C57BL/KaLwRij mice (Figure 4A). Successful tumor engraftment was verified by immunohistochemical staining, which revealed extensive infiltration of CD138^+^ plasma cells in the marrow space (Figure 4B). This was further corroborated by a >1500‐fold increase in luciferase mRNA expression in the tumor‐bearing bone marrow compared to normal controls (Figure 4C). Once disease establishment was confirmed, the therapeutic regimen was initiated (Figure 4D). Serial bioluminescence imaging revealed rapid tumor expansion in the PBS and DC Exo groups, with signals disseminating beyond the primary injection site. In stark contrast, DB Exo treatment significantly arrested tumor progression (Figure 4E). Quantitative analysis of the photon flux confirmed that while DC Exo failed to restrain tumor growth, DB Exo reduced the tumor burden by approximately 72% compared to the control group (Figure 4F). The treatment was well‐tolerated, with all mice maintaining stable body weight throughout the study (Figure 4G). Necropsy revealed marked splenomegaly in both exosome‐treated groups compared to controls (Figure 4H). Quantitative measurement confirmed an increase in average spleen weight from approximately 130 mg in the control mice to approximately 340 mg in both the DC Exo and DB Exo groups (Figure 4I). Interestingly, while splenomegaly suggests a systemic immune response or reticuloendothelial engagement triggered by exosome administration, only DB Exo translated this systemic modulation into effective antitumor immunity.

### 2.5 DB Exo Coordinate Systemic Immune Priming and Cytotoxic T Cell Expansion

**FIGURE 5:**
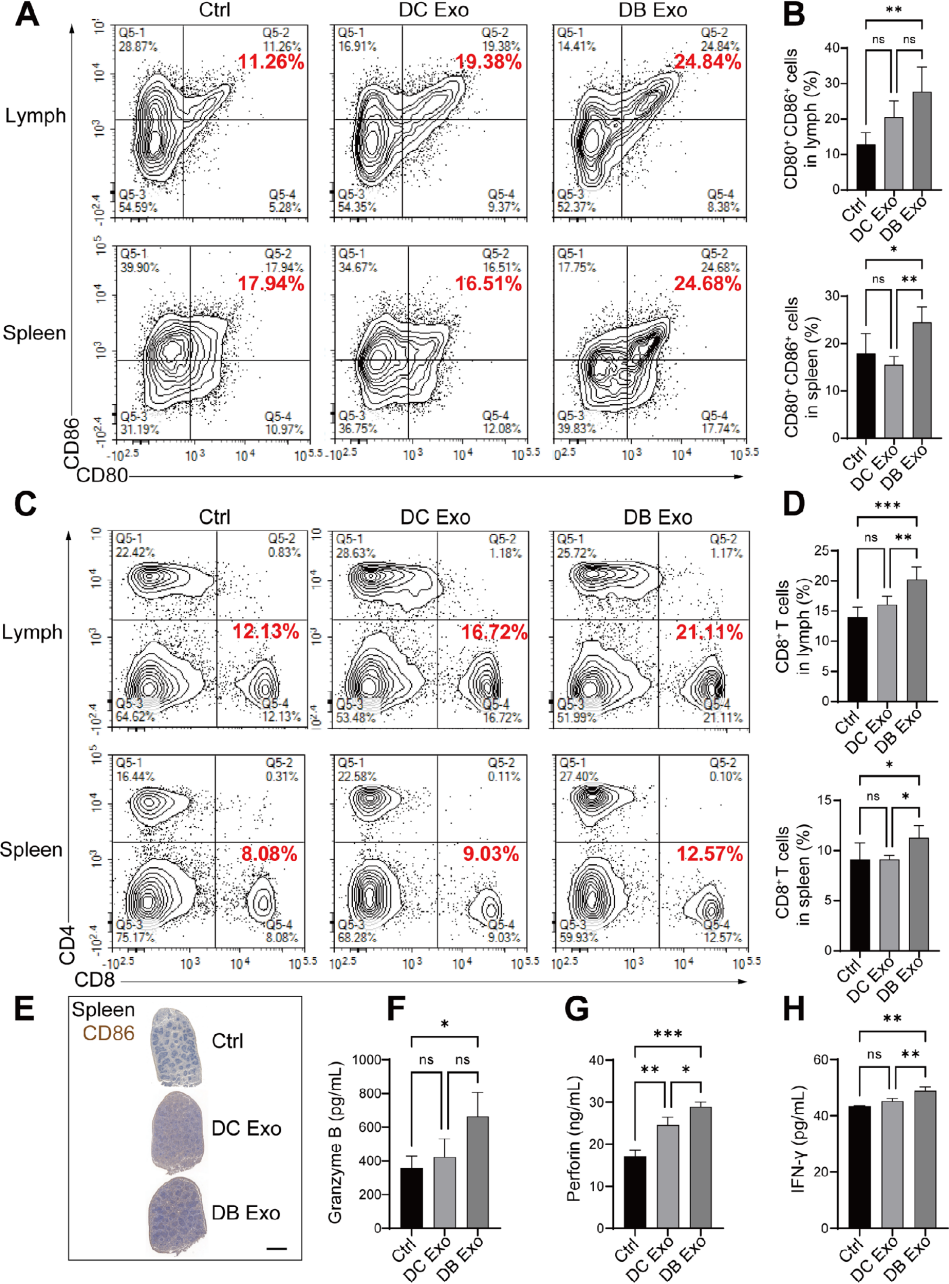
**DB Exo promote dendritic cell maturation and systemic CD8^+^ T cell activation in secondary lymphoid organs**. (A) Representative flow cytometry contour plots showing CD80 and CD86 expression in dendritic cells from lymph nodes and spleens. (B) Quantitative analysis of the frequency of mature DCs in lymph nodes and spleens. *n =* 6. (C) Representative flow cytometry analysis of CD4^+^ and CD8^+^ T cell subsets in lymph nodes and spleens. (D) Quantification of the percentage of CD8^+^ T cells in lymph nodes and spleens. *n =* 6. (E) Immunohistochemical staining of CD86 in splenic tissue sections from treated mice. Scale bar: 50 µm. (F–H) ELISA measurement of serum concentrations of Granzyme B (F), Perforin (G), and IFN‐γ (H). *n =* 6. Statistical significance was analyzed using one‐way ANOVA followed by Tukey's multiple comparisons test for panels B, D, F, G, and H. All data are presented as mean ± SD. ^*^ *p* < 0.05, ^**^ *p* < 0.01, ^***^ *p* < 0.001, ns: not significant.

To elucidate the immunological mechanisms underlying the observed splenomegaly and therapeutic efficacy, we performed comprehensive immunoprofiling of the secondary lymphoid organs. Given that the spleen and draining lymph nodes serve as the primary sites for antigen presentation and adaptive immune initiation, we focused on these tissues to evaluate systemic immune mobilization. The generation of robust antitumor immunity necessitates the activation of DCs to effectively prime T cells. We first evaluated DC maturation status by quantifying the expression of costimulatory molecules. Flow cytometric profiling revealed that DB Exo administration significantly promoted the maturation of the DC compartment. In the lymph nodes, the frequency of mature DCs (defined as CD80^+^CD86^+^ gated on CD11c^+^ cells) increased significantly from 11.26% ± 1.4% in PBS‐treated control mice to 24.84% ± 3.2% in the DB Exo‐treated group (Figure 5A,B). We next investigated whether this enhanced DC antigen‐presenting capacity translated into the expansion of cytotoxic T lymphocytes (CTLs), the ultimate effectors of myeloma clearance. Consistent with the DC maturation data, DB Exo treatment triggered a significant expansion of the CD8^+^ T cell population (Figure 5C,D). In the lymph nodes and spleen, the CD8^+^ T cell frequency increased to 21.11% and 12.57%, respectively, representing a significant elevation compared to both the PBS and DC Exo groups (Figure 5C,D). This suggests that DB Exo convert the immunosuppressive state into an immunogenic phenotype capable of supporting T cell proliferation. This enhanced activation in the splenic microenvironment was further corroborated by immunohistochemical staining, which showed dense clusters of CD86^+^ cells within the splenic follicles of DB Exo‐treated mice (Figure 5E). Finally, to assess the functional activity of these expanded effectors, we quantified systemic levels of key cytotoxic cytokines. ELISA analysis of serum demonstrated that DB Exo treatment induced a coordinated upregulation of effector molecules, including Granzyme B (Figure 5F), Perforin (Figure 5G), and the type‐1 cytokine IFN‐γ (Figure 5H). Notably, while DC Exo induced partial elevation of Perforin, only DB Exo achieved a simultaneous and significant induction of all three markers, confirming that the dual‐function strategy is essential for establishing a fully functional cytotoxic environment. Collectively, these data indicate that DB Exo suppress myeloma progression by orchestrating a systemic immune response characterized by robust DC maturation and the subsequent generation of functional cytotoxic T cells.

### 2.6 DB Exo Suppress Subcutaneous Tumor Growth and Maintain a Favorable Biosafety Profile

**FIGURE 6:**
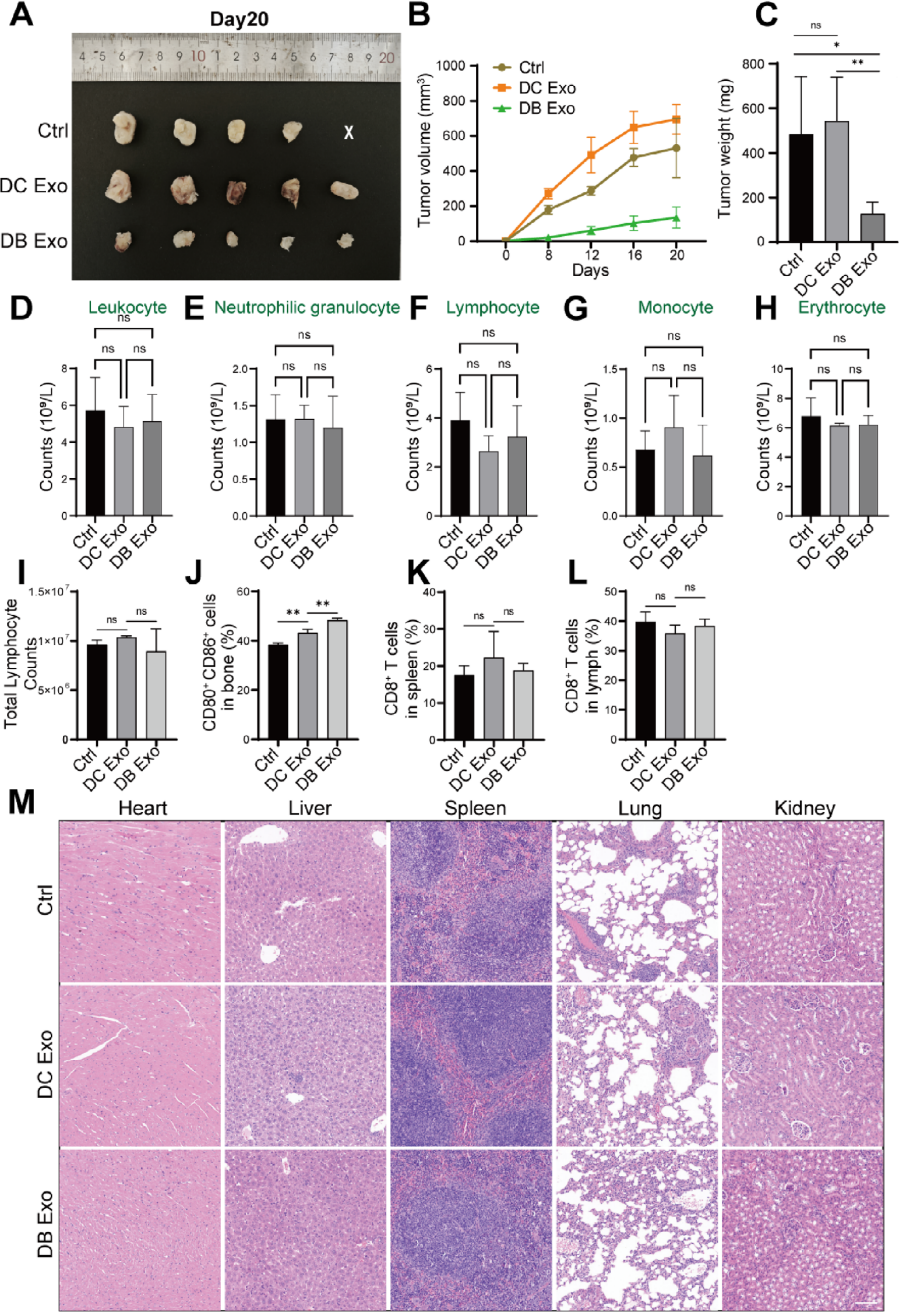
**Antitumor efficacy in a subcutaneous model and systemic biosafety evaluation**. (A–C) Evaluation of therapeutic efficacy in a subcutaneous 5TGM1 model. *n =* 6. (A) Photographic images of tumors excised at the study endpoint (Day 20). Note: ‘X’ indicates a sample excluded from the final tumor weight analysis because the mouse reached the humane endpoint due to excessive tumor burden prior to Day 20. (B) Tumor growth curves measuring tumor volume (mm^3^) over time in mice treated with PBS, DC Exo, or DB Exo. (C) Weight (mg) of excised tumors at the endpoint. (D–M) Biosafety assessment in healthy (non‐tumor‐bearing) mice. (D–H) Complete blood count (CBC) analysis showing levels of leukocytes (D), neutrophils (E), lymphocytes (F), monocytes (G), and erythrocytes (H). *n =* 6. (I–L) Flow cytometric analysis of total lymphocyte counts (I), mature DCs (CD80^+^CD86^+^) in bone marrow (J), and T cell subsets in spleen (K) and lymph nodes (L). *n =* 6. (M) Representative hematoxylin and eosin (H&E) staining of major organs (heart, liver, spleen, lung, kidney) showing preserved tissue architecture. Scale bar: 50 µm. Statistical significance was analyzed using one‐way ANOVA followed by Tukey's multiple comparisons test for panels C‐L. All data are presented as mean ± SD. ^*^ *p* < 0.05, ^**^ *p* < 0.01, ns: not significant.

To validate the robustness of the therapeutic efficacy beyond the orthotopic niche, we evaluated DB Exo in a subcutaneous 5TGM1 plasmacytoma model. Following the establishment of palpable tumors, mice were treated according to the established regimen. Consistent with the orthotopic data, DB Exo treatment exerted potent tumor suppression. While tumors in the PBS and DC Exo groups grew aggressively, reaching volumes of >500 mm^3^, the DB Exo group showed significantly slowed growth kinetics (Figure 6A,B). At the study endpoint (Day 20), the excised tumor weight in the DB Exo group was reduced by approximately 75% compared to controls (Figure 6C), confirming that the anti‐myeloma efficacy of DB Exo is robust across different physiological microenvironments.

Subsequently, to ensure the translational potential of the platform, a biosafety assessment was conducted in healthy C57BL/KaLwRij mice. This evaluation aimed to verify that DB Exo administration does not induce off‐target toxicity or aberrant immune dysregulation in the absence of tumor burden. The quantification of key blood cell populations, including leukocytes, neutrophils, monocytes, and erythrocytes, revealed no statistically significant deviations from physiological ranges, indicating no evidence of myelosuppression or hemolysis (Figure 6D–H). Similarly, total lymphocyte counts in lymph nodes were comparable across groups, ruling out non‐specific lymphoid depletion or hyperplasia (Figure 6I).

To verify the immunological specificity of the treatment, we profiled immune subsets in healthy tissues. Interestingly, DB Exo retained their ability to accumulate in the bone marrow and activate antigen‐presenting cells, as evidenced by a significant upregulation of CD80/CD86 on bone marrow dendritic cells (Figure 6J). However, unlike the marked expansion of CD8^+^ T cells observed in tumor‐bearing mice (Figure 5), DB Exo treatment in healthy mice did not induce significant CD8^+^ T cell expansion in the spleen or lymph nodes (Figure 6K,L). This context‐dependent response suggests that DB Exo provide costimulatory and ligand‐scavenging functions but rely on tumor‐derived cues in the microenvironment to drive cytotoxic T cell expansion, indicating a tumor‐conditional activation profile that minimizes the risk of systemic autoimmunity in healthy tissues. Histological examination of major organs (heart, liver, spleen, lung, kidney) revealed no pathological alterations, fibrosis, or inflammatory infiltrates (Figure 6M), collectively confirming a robust safety profile with minimal systemic toxicity.

## 3 Discussion

Multiple myeloma (MM) remains a persistent malignancy characterized by repeated cycles of remission and relapse, largely sustained by the protective bone marrow microenvironment (BMM) [35, 36]. This niche not only supports plasma cell survival through cytokine signaling but also promotes local immune evasion [35]. In this study, a dendritic cell‐derived exosome platform (DB Exo) was developed to surface display BCMA, functioning as a dual‐action therapeutic to sequester survival ligands and stimulate host immunity. The results demonstrate that DB Exo effectively modulate both the cytokine environment and the cellular composition within the BMM, leading to strong antitumor efficacy across various murine MM models.

The therapeutic effect of DB Exo begins with the disruption of the APRIL/BAFF‐BCMA signaling axis, on which MM cells critically depend for survival and proliferation [14]. By displaying BCMA on the exosomal surface, DB Exo function as molecular decoys to intercept these ligands before they engage tumor‐expressed receptors. The observation that DB Exo treatment leads to a near‐complete depletion of free APRIL (>98%) and a partial reduction in BAFF (∼35%) reflects the well‐documented differential binding affinity of BCMA for its two cognate ligands. Biophysical measurements using monomeric receptors have established that BCMA binds APRIL with a K_D of approximately 16 nm, whereas its affinity for BAFF is approximately 100‐fold lower (K_D ∼1.6 µm) [37]. This intrinsic selectivity is consistent with the physiological role of the BCMA–APRIL axis as the primary signaling module for plasma cell survival, while BAFF preferentially engages BAFFR on earlier B cell stages [38]. Accordingly, the observed clearance disparity is a predictable biophysical consequence of displaying wild‐type BCMA on the exosomal surface, rather than a limitation of the platform. Notably, a recent study engineering soluble BCMA decoy receptors confirmed that wild‐type sBCMA‐Fc effectively blocked APRIL signaling but required a 500‐fold affinity enhancement through directed evolution to achieve equivalent BAFF neutralization, further validating that the ∼35% BAFF attenuation achieved by DB Exo represents the ceiling of wild‐type BCMA‐mediated BAFF clearance [39]. After establishing that the differential clearance reflects intrinsic receptor selectivity, we examined the functional sufficiency of near‐complete APRIL neutralization. In APRIL‐stimulated cells, DB Exo treatment reduced p52 from 1.6 to 0.9 and pIκBα from 2.2 to 0.6, demonstrating that near‐complete APRIL sequestration is sufficient to suppress both canonical and non‐canonical NF‐κB survival signaling. These findings validate that disrupting the ligand‐receptor signaling axis is sufficient to suppress tumor survival, supporting “ligand starvation” as an effective therapeutic strategy.

Beyond validating the ligand starvation strategy, the competitive inhibition assay using BCMA‐overexpressing 5TGM1 cells revealed that the therapeutic potency of DB Exo scales with target antigen density. In clinical practice, BCMA is broadly but heterogeneously expressed on malignant plasma cells, with high‐density expression and elevated soluble BCMA levels often correlating with advanced disease stages and poor outcomes in relapsed/refractory MM [13]. The 5TGM1‐BCMA model effectively recapitulates this high‐antigen‐density phenotype, providing a stringent and clinically representative context for evaluating the decoy mechanism. Notably, DB Exo exhibited superior inhibitory activity against BCMA‐overexpressing cells compared to wild‐type counterparts. This finding suggests a dependency‐driven vulnerability, whereby cells with higher BCMA expression are more susceptible to DB Exo‐mediated ligand deprivation due to their heightened reliance on APRIL/BAFF survival signaling. Conversely, the dependence of the decoy mechanism on ligand‐receptor stoichiometry raises the question of therapeutic efficacy when BCMA expression is reduced. In clinical settings, BCMA downregulation through gamma‐secretase‐mediated shedding or biallelic loss has been reported as a resistance mechanism to BCMA‐directed therapies [19, 20]. Under such conditions, the ligand‐scavenging function of DB Exo would remain intact because it targets soluble APRIL and BAFF irrespective of tumor surface BCMA levels, but the preferential growth‐inhibitory effect observed in high‐BCMA clones would diminish. Tumors that have escaped BCMA‐dependent survival signaling altogether, for instance through compensatory activation of alternative pathways such as IL‐6/JAK‐STAT or adhesion‐mediated drug resistance, would be less susceptible to ligand deprivation alone [8, 40]. In this context, the immune activation arm of DB Exo, mediated by costimulatory molecules and DC priming, becomes the primary therapeutic mechanism. Therefore, combining DB Exo with agents that restore or stabilize surface BCMA, such as gamma‐secretase inhibitors, or with checkpoint inhibitors that potentiate the T cell response, may extend therapeutic coverage to BCMA‐low or BCMA‐negative resistant populations.

Relative to our prior HEK‐293T‐derived BCMA nanovesicles (Re‐BCMA‐NVs), which functioned exclusively as passive cytokine decoys, DB Exo achieve dual functionality by anchoring the same decoy receptor onto a dendritic cell membrane scaffold [26]. This specific integration of an immunostimulatory vesicle scaffold with a decoy receptor distinguishes DB Exo from other emerging exosome therapeutics. While engineered exosomes have been used effectively as vehicles for targeted delivery of chemotherapeutics like bortezomib or as cytotoxic effectors derived from CAR‐T cells, these approaches have primarily focused on direct tumor killing [41, 42]. In contrast, DB Exo are designed to function as molecular decoys that remodel the immunosuppressive signaling environment while simultaneously harnessing the natural antigen‐presenting capacity of DCs. The rationale for selecting this DC‐derived scaffold is supported by our observation that unmodified DC exosomes (DC Exo) retained minor antitumor activity and induced basal T cell activation within the immunosuppressive orthotopic niche. Unlike synthetic carriers that serve as passive vehicles, DC exosomes possess intrinsic immunostimulatory properties that are not completely silenced by the tumor microenvironment [43]. However, their failure to control subcutaneous tumors confirms that this bioactivity, while superior to inert materials, is insufficient on its own and requires targeted receptor engineering to achieve potent, systemic efficacy. DB Exo fulfilled this requirement by exerting a second, distinct therapeutic action beyond ligand scavenging: directly reshaping the immunological landscape of the bone marrow and secondary lymphoid organs with an immunostimulatory capacity that markedly surpassed that of unmodified DC Exo. DB Exo treatment induced substantial increases in CD80^+^CD86^+^ mature dendritic cell frequencies and a subsequent expansion of CD8^+^ T cell populations, together indicating a transition from an immunosuppressive to an immune‐active state. This immunostimulatory effect likely stems from the natural cargo of DC‐derived exosomes, which carry peptide‐MHC complexes and costimulatory molecules capable of transferring activation signals to endogenous antigen‐presenting cells or directly priming T cells [31]. This capacity is particularly relevant in MM, where the profoundly immunosuppressive BMM impairs host DC function and renders conventional DC exosome‐based vaccines largely ineffective [44, 45]. DB Exo thus serve as an alternative route, delivering DC‐like stimulatory signals into an environment where endogenous antigen presentation has been compromised. While the data demonstrate elevated effector cytokine production and cytotoxic marker expression, the antigen specificity of the ensuing T cell response was not resolved at the receptor level. Whether tumor‐specific clones are preferentially expanded or whether the response reflects broader, non‐specific immune mobilization remains an important open question, addressable in future studies through MHC‐tetramer staining or TCR sequencing.

Effective engagement of the bone marrow compartment is central to achieving meaningful therapeutic responses in MM [46]. Biodistribution analysis revealed that DB Exo achieved significantly greater bone accumulation than unmodified DC Exo. Although this enhanced tropism may arise from multiple factors, including phenotypic changes in the parental cells following lentiviral transduction and the resulting alterations in exosomal surface composition, the displayed BCMA domain likely contributes by engaging APRIL, which is locally produced by osteoclasts and macrophages within the myeloma niche [14]. Such interactions could preferentially retain DB Exo in the marrow compartment, analogous to the organotropic homing observed with exosomal integrins in other disease settings [47]. Nevertheless, the current biodistribution analysis, based on endpoint fluorescence imaging, does not provide high‐resolution pharmacokinetic data. Determining the precise percentage of the injected dose that reaches the bone marrow relative to sequestration by the liver and other clearance organs, and dissecting the relative contributions of BCMA and other surface alterations to this enhanced tropism, remain important objectives for future investigation.

Despite these pharmacokinetic uncertainties, the demonstrated ability of DB Exo to access the bone marrow niche and elicit local immune remodeling provides a foundation for considering their translational feasibility. From a manufacturing and safety standpoint, DB Exo offer several advantages over traditional cell‐based immunotherapies. The use of an engineered, allogeneic DC line as a production source enables standardized manufacturing and avoids the functional impairments often found in autologous DCs from heavily pretreated MM patients [48]. Moreover, this strategy avoids the safety concerns associated with myeloma cell‐derived exosome vaccines [49, 50]. While strategies involving HSP70‐engineered tumor exosomes have shown potential in priming NK and T cells, the use of tumor‐derived vesicles carries the risk of transferring oncogenic factors or immunosuppressive contents [51, 52, 53]. By contrast, DB Exo are derived from professional antigen‐presenting cells, ensuring a favorable immunogenic profile without contamination by tumor‐promoting signals. Furthermore, the absence of overt systemic toxicity or cytokine release‐like symptoms in immunocompetent models is encouraging. However, clinical translation requires monitoring whether the high‐density display of engineered BCMA triggers neutralizing antibodies that accelerate drug clearance upon repeated dosing. Furthermore, the preferential uptake of DB Exo by antigen‐presenting cells minimizes the risk of the decoy acting as a ligand reservoir by ensuring that sequestered survival factors are degraded rather than accumulating in the circulation.

There are several limitations to this study. The experimental timeframe focused on immediate tumor suppression and did not evaluate the induction of long‐term immune memory or the prevention of tumor recurrence. Additionally, while CD8^+^ T cell expansion was strong, the clonal diversity and antigen specificity of these cells remain to be verified. The reliance on syngeneic and cell line‐based models, while necessary for mechanistic studies, does not fully capture the genetic and clinical heterogeneity of human disease. Validation in patient‐derived xenograft (PDX) systems will be a necessary step in confirming the platform's broad applicability. Finally, given the potent immunomodulatory effects observed, exploring rational combinations with immune checkpoint blockade or other immunomodulatory agents represents a promising area for further research.

Future studies could extend this receptor‐displaying exosome platform to other ligand‐dependent diseases. For instance, in systemic lupus erythematosus (SLE), where APRIL and BAFF drive the survival of autoreactive B cells, DB Exo could function as a systemic decoy to reset B cell homeostasis [54]. Furthermore, by substituting the surface‐displayed receptor, this platform could be adapted to neutralize inhibitory factors such as TGF‐β or VEGF in solid tumor microenvironments. This adaptable design, combined with the low immunogenicity and natural stability of exosomes, offers a promising path for the development of exosome‐based nanomedicines capable of modulating the immune state in complex disease conditions.

## 4 Conclusion

This study establishes a modular, cell‐free platform by engineering dendritic cell‐derived exosomes to surface display BCMA. These DB Exo neutralize the growth factors APRIL/BAFF, thereby disrupting NF‐κB‐mediated survival signaling, while simultaneously stimulating dendritic cell maturation and cytotoxic T cell expansion. In vivo, the platform effectively suppressed tumor progression in both orthotopic and extramedullary models with no observable systemic toxicity. These data demonstrate that decoy‐receptor‐functionalized exosomes represent a robust strategy to remodel the immunosuppressive microenvironment and eliminate malignant niches in multiple myeloma.

## 5 Experimental Section

### 5.1 Cell Lines and Cell Culture

DC2.4 (mouse dendritic cell line) and HEK‐293T (human embryonic kidney cell line) cells were purchased from the American Type Culture Collection (ATCC). 5TGM1 (mouse MM cell line) and 5TGM1‐Luc (mouse MM cell line) were purchased from Cellcook (Guangzhou, China). Cells were cultured in appropriate media: DC2.4 and HEK‐293T cells in DMEM (Gibco), and 5TGM1/5TGM1‐Luc cells in IMDM (Gibco), each supplemented with 10% fetal bovine serum (ExCell Bio). All cells were incubated at 37°C in a humidified atmosphere containing 5% CO_2_.

### 5.2 Reagents and Antibodies

WGA 350 (W11263) was purchased from Thermo Fisher Scientific. Anti‐GFP antibody (AE078) was purchased from ABclonal. Anti‐ALIX (382205), Anti‐CD63 (R23327), and Anti‐TSG101 (381538) antibodies were purchased from Zen‐Bio. Anti‐ACTIN (P30002), Anti‐pIκBα (TP56280), Anti‐CD138 (M069249), Anti‐CD86 (PU885801), HRP‐conjugated goat anti‐mouse IgG (M21001), and HRP‐conjugated goat anti‐rabbit IgG (M21002) antibodies were purchased from Abmart. Anti‐p100/p52 antibody (AF6373) was purchased from Affinity Biosciences. CFSE Cell Division Tracker Kit (423801), purified anti‐mouse CD3ε (100340) and purified anti‐mouse CD28 (102116) were purchased from BioLegend. Mouse GM‐CSF (HA210930) and mouse IL‐4 (HA210902) were purchased from HuaBio. D‐Luciferin potassium salt (115144‐35‐9) was purchased from GLPBio. Sulfo‐Cyanine5 (Cy5) dye (HY‐D0821) was purchased from MCE.

### 5.3 Plasmids and Stable Cell Lines

pCDH‐CMV‐MCS‐EF1‐puro and pCDH‐CMV‐Mus‐BCMA‐EGFP‐EF1‐puro plasmids were obtained from MiaoLing Bio, China. To obtain stable cell lines, the target plasmids were transfected into HEK‐293T cells using PEI 25K (Aladdin, China) and the supernatant was harvested at 48 and 72 h. DC2.4 and 5TGM1 cells were then infected with lentivirus and selected with puromycin (0.5 µg/mL) to obtain stable cell lines.

### 5.4 Exosome Isolation and Purification

Exosomes were isolated from the cell culture supernatant using differential centrifugation. Culture media were collected from DC2.4, DB, or 5TGM1 cells. The medium was centrifuged at 300 × g for 10 min to remove cells, followed by 2000 × g for 10 min to remove dead cells. Subsequently, the supernatant was centrifuged at 10 000 × g for 30 min to remove cell debris. All steps were performed at 4°C. Finally, the supernatant was ultracentrifuged at 110 000 × g for 70 min (Optima XPN‐100, Beckman Coulter) to pellet the exosomes. The exosome pellet was washed with PBS and centrifuged again at 110 000 × g for 70 min. The final exosomes were resuspended in PBS and stored at −80°C.

### 5.5 Exosome Characterization

For transmission electron microscopy (TEM), 10 µL of the exosome solution was loaded onto a copper grid. After 1 min, the grid was stained with 2% uranyl acetate for 1 min and allowed to dry. Images were acquired using a JEM‐1400Flash transmission electron microscope (JEOL, Japan). For nanoparticle tracking analysis (NTA), exosomes were diluted with PBS. Particle size and concentration were measured using a NanoSight instrument (Malvern Panalytical, UK) and analyzed with NTA 3.4 software. For zeta potential analysis, exosomes were diluted in PBS, and the surface charge was measured using a Malvern Zetasizer Pro (Malvern Panalytical, UK).

### 5.6 Nano‐Flow Cytometry Analysis

Surface proteins on single exosomes were detected using a Flow NanoAnalyzer (NanoFCM Inc., Xiamen, China). Exosomes were mixed with fluorescent antibodies: FITC anti‐mouse CD86 (BioLegend, 105005) and APC anti‐mouse MHC‐II (BioLegend, 107614). The mixture was incubated at 37°C for 30 min in the dark. Subsequently, exosomes were washed with PBS to remove free antibodies. Finally, the samples were analyzed on the Flow NanoAnalyzer.

### 5.7 Isolation and Culture of Primary Cells

Bone marrow cells were harvested from the femurs and tibias of C57BL/6 mice under sterile conditions. After red blood cell lysis with RBC lysis buffer (Solarbio Life Sciences, R1010), the remaining cells were washed and resuspended in complete RPMI‐1640 medium supplemented with 10% fetal bovine serum (FBS), 1% penicillin‐streptomycin, GM‐CSF (20 ng/mL) and IL‐4 (10 ng/mL). Cells were plated in non‐tissue‐culture‐treated Petri dishes at a density of 1 × 10^6^ cells/mL. On day 3, half of the culture medium was replaced with fresh medium containing the same concentration of cytokines. On day 5, loosely adherent and non‐adherent cells were collected and used as immature BMDCs for downstream experiments. For CD3^+^ T cell isolation, 48‐well plates were coated one day prior with 200 µL per well of PBS containing 2 µg anti‐mouse CD3ε antibody, and incubated at 4°C overnight. Spleens from C57BL/6 mice were harvested, mechanically dissociated and passed through a 70 µm cell strainer, and centrifuged at 300 × g for 5 min. Red blood cells were lysed with 3 mL RBC lysis buffer on ice for 2 min. Cells were washed twice with MojoSort Buffer and counted. For magnetic negative selection, cells were incubated with Biotin‐Antibody Cocktail (10 µL per 1 × 10^7^ cells) for 15 min on ice, followed by Streptavidin Nanobeads (10 µL per 1 × 10^7^ cells) for 15 min. After adding 2.5 mL MojoSort Buffer, tubes were placed on a magnetic stand, and the unbound unlabeled cell fraction was collected twice and pooled. Cells were centrifuged, and the final CD3^+^ T cell pellet was resuspended in complete medium for downstream use.

### 5.8 Ligand Sequestration Assay

To evaluate the ability of DB Exo to sequester APRIL and BAFF, exosomes (50 µg) were mixed with cell culture medium containing soluble APRIL or BAFF (derived from 5TGM1 culture supernatant or recombinant protein). The mixture was incubated at 37°C for 2 h to facilitate binding. Following incubation, the mixture was ultracentrifuged at 110 000 × g for 70 min to pellet the exosomes along with bound ligands. The supernatant was collected, and the residual concentrations of ligands were quantified using specific ELISA kits: Mouse BAFF ELISA Kit (Abcam, ab119580) and Mouse TNFSF13/APRIL ELISA Kit (FineTest, EM2284).

### 5.9 CFSE Proliferation Assay

Cells were pelleted and resuspended at 1 × 10^7^ cells/mL in CFSE working solution (5 µm), then incubated for 20 min at room temperature in the dark. Staining was quenched by adding five volumes of complete medium containing 10% FBS. Cells were then pelleted, resuspended in pre‐warmed complete medium, incubated for an additional 10 min, washed, and used for downstream applications. Proliferation was quantified as the percentage of CFSE‐low cells gated relative to an undivided reference population at time zero, determined by flow cytometry.

### 5.10 Cellular Uptake Assay

DC Exo (50 µg) and DB Exo (50 µg) were labeled with 100 µL of 2 µm Cy5 dye at 4°C for 6 h. Free dye was removed by centrifugal ultrafiltration at 300 × g for 20 min. For confocal microscopy, Cy5‐labeled exosomes were incubated with BMDCs at 37°C for 6 h. Cells were washed three times with PBS, fixed with 4% paraformaldehyde fixative (Biosharp, BL539A) for 10 min at room temperature, and stained with 2 µm DAPI (Solarbio Life Sciences, 28718‐90‐3) for 10 min. Images were acquired using a confocal laser scanning microscope (LSM880, Zeiss). For flow cytometry analysis, Cy5‐labeled exosomes were incubated with DC2.4 cells and 5TGM1‐Luc cells at 37°C for 12 h. After incubation, non‐adherent 5TGM1‐Luc cells in the supernatant were collected; adherent DC2.4 cells were washed with PBS and detached with 0.25% trypsin (GIBCO, 15050057). Both cell populations were centrifuged at 300 × g for 5 min and analyzed by flow cytometry (NovoCyte, Agilent) to determine exosome uptake rates.

### 5.11 Cell Line Construction and Proliferation Assay

The 5TGM1‐BCMA stable cell line was established by lentiviral transduction of parental 5TGM1 cells with a pCDH‐CMV‐Mus‐BCMA‐EGFP‐EF1‐puro construct, followed by selection with 0.5 µg/mL puromycin. For viability and proliferation assessments, the Cell Counting Kit‐8 (CCK‐8) was used according to the manufacturer's instructions. To evaluate the therapeutic effect of DB Exo, 5TGM1 cells (3000 cells/well) were seeded in 96‐well plates and treated with exosomes for 48 h. To assess the impact of BCMA overexpression on baseline growth, wild‐type 5TGM1 and 5TGM1‐BCMA cells were seeded at an equal density, with absorbance at 450 nm measured at 24, 48, and 72 h.

### 5.12 Western Blotting

Cells were lysed in lysis buffer (Epizyme Biotech, PC101) containing protease inhibitor (Epizyme Biotech, GRF101). Protein concentration was quantified using the Bradford assay (Bio‐Rad, 5000201). The protein samples were separated by 10%–12% SDS‐PAGE and transferred to PVDF membranes (Bio‐Rad, 1620177). The membranes were then blocked with 5% skimmed milk and incubated with primary antibodies. Signals were detected using an imaging system (Baygene Biotech, BG‐gdsAUTO 730) at room temperature.

### 5.13 RNA Isolation and Quantitative Reverse Transcription PCR (RT‐qPCR)

Total RNA was extracted using an RNA extraction reagent (Vazyme, R401‐01). cDNA was synthesized using cDNA Synthesis SuperMix (TransGen Biotech, AE341‐02). RT‐qPCR was performed using SYBR Green RT‐qPCR SuperMix (TransGen Biotech, AQ101‐01) on a LightCycler 96 Instrument (Roche). Relative mRNA expression fold changes were calculated using the 2^−ΔΔCt^ method. The sequences of the RT‐qPCR primers are provided in the Supporting Information (Table S1).

### 5.14 Co‐Culture of Mouse Spleen or Bone Marrow Cells With Tumor Cells

Single‐cell suspensions of spleen or bone marrow from C57BL/6 mice were prepared under sterile conditions, followed by RBC lysis and resuspension in complete RPMI‐1640 medium. Spleen or bone marrow cells were pretreated with exosomes for 12 h. After incubation, exosome‐treated spleen or bone marrow cells were co‐cultured with 5TGM1‐Luc cells at a 10:1 ratio and imaged on day 0 to record the initial tumor cell proportion. After 48 h of co‐culture, the proportion of tumor cells was assessed by image analysis, supernatants were collected for cytokine quantification by ELISA, and remaining cells were analyzed by flow cytometry to evaluate immune cell composition.

### 5.15 ELISA

For sample preparation, cell culture supernatants were centrifuged at 500 × g for 10 min to remove cellular debris. Mouse blood samples were centrifuged at 300 × g for 20 min to collect serum. Concentrations of IFN‐γ (Elabscience, M0048), TNF‐α (Elabscience, M3063), Granzyme B (Elabscience, M0594), and Perforin 1 (Elabscience, M0890) in cell culture supernatants and serum were measured using the corresponding ELISA kits according to the manufacturer's instructions.

### 5.16 Flow Cytometry Analysis

To assess DC maturation, cells were stained with FITC anti‐mouse CD11c (BioLegend, 117306), BV421 anti‐mouse CD80 (BioLegend, 104725) and APC anti‐mouse CD86 (BioLegend, 105012) for 15 min in the dark. To evaluate T cell activation, cells were stained with FITC anti‐mouse CD3 (BioLegend, 100204), APC anti‐mouse CD4 (BioLegend, 100516) and BV421 anti‐mouse CD8 (BioLegend, 100753) for 15 min in the dark.

### 5.17 Immunofluorescence Assay (IF)

The cell suspension was cytocentrifuged onto a glass slide. Cells were fixed with 4% paraformaldehyde for 15 min at room temperature and washed three times with PBS for 5 min each. The cells were then permeabilized with 0.5% Triton X‐100 for 10 min at room temperature, washed three times with PBS, and blocked with 10% goat serum for 45 min at room temperature. The corresponding primary antibody was added and incubated overnight at 4°C. After three washes with PBST, the fluorescent secondary antibody was added and incubated for 1 h at room temperature protected from light. Finally, an anti‐fade mounting medium was applied, and the slides were imaged promptly. Images were acquired using a Zeiss LSM 880 confocal microscope. Image processing and analysis were performed using ZEN software (Carl Zeiss).

### 5.18 Animal Studies

All animal experiments were performed in accordance with institutional guidelines and were approved by the Institutional Animal Care and Use Committee of Sun Yat‐sen University (SYSU‐IACUC‐2024‐000116).

For safety studies in normal C57BL/6 mice, exosomes were intravenously administered daily for 1 week. Subsequently, blood samples were collected into EDTA tubes for hematological analysis using an automated analyzer (BC‐5000Vet, Mindray Animal Medical, China), while lymph nodes, spleens, and bone marrow were harvested and processed into single‐cell suspensions for flow cytometric analysis of DC and T cell activation.

For the biodistribution study, exosomes were incubated with Cy5 dye at 4°C for 6 h. Excess dye was removed using centrifugal ultrafiltration. Labeled exosomes were then administered to mice via tail vein injection. At 12 h post‐injection, lymph nodes, heart, liver, spleen, lungs, kidneys, and femurs were harvested for fluorescence imaging. To establish an orthotopic MM mouse model, 5TGM1‐Luc cells were injected into the femoral cavity of C57BL/KaLwRij mice. Mice were then randomly assigned to three groups. From day 3 post‐implantation, exosomes were administered intravenously at a dose of 10 mg/kg every 3 days for a total of 5 doses. Bioluminescence imaging was performed every 5 days to monitor tumor progression. For each bioluminescence imaging session, D‐luciferin potassium salt was administered via tail vein injection at a dose of 100 mg/kg, and mice were imaged 10 min post‐injection to allow optimal substrate distribution and signal stabilization. Images were acquired using a PerkinElmer IVIS Spectrum in vivo imaging system and analyzed with Living Image software. The tumor burden was quantified by drawing standardized regions of interest (ROIs) over the tumor‐bearing femur, with the bioluminescence intensity expressed as average radiance (p/s/cm^2^/sr). On day 15, mice were euthanized, and lymph nodes, spleens, and bone marrow were collected for flow cytometric analysis.

For the subcutaneous MM mouse model, mice were randomly divided into three groups. 5TGM1 cells (5 × 10^6^ cells/mouse) were subcutaneously injected into the right flank of C57BL/KaLwRij mice. Tumor growth and body weight were recorded daily. Tumor volume was calculated using the formula (Length × Width^2^) / 2. Mice in the DC Exo and DB Exo groups were intravenously injected with exosomes at 10 mg/kg, while control mice received PBS. All mice were sacrificed at the experimental endpoint. In all experiments, the subcutaneous tumor volumes did not exceed 1000 mm^3^.

### 5.19 Immunohistochemistry (IHC) and H&E Staining

Tissue sections were deparaffinized, rehydrated, and subjected to heat‐induced antigen retrieval. Sections were blocked with 10% goat serum for 30 min, then incubated with primary antibodies against CD138 or CD86 overnight at 4°C, followed by incubation with HRP‐conjugated secondary antibody and DAB chromogen development. For hematoxylin and eosin (H&E) staining, sections were stained according to standard protocols. All histological sections were examined and imaged using a Nikon INTENSILIGHT C‐HGFI upright fluorescence microscope.

### 5.20 Statistical Analysis

Data were analyzed using GraphPad Prism software (version 9.5.0, San Diego, CA, USA). For Western blot densitometry, band intensities were normalized to the β‐Actin loading control, and relative protein levels were expressed as fold change relative to the control group (set to 1.0). For RT‐qPCR, relative mRNA expression was calculated using the 2^−ΔΔCt^ method and normalized to the control group (set to 1.0). One sample in the subcutaneous tumor model (Figure 6A) was excluded because the mouse reached the humane endpoint before the study conclusion. Comparisons between two groups were performed using the unpaired two‐tailed Student's t‐test. For comparisons among more than two groups, one‐way analysis of variance (ANOVA) followed by Tukey's multiple comparisons test was utilized. Dunnett's test was used to compare each treatment concentration with the zero‐concentration control. The sample size (*n*) for each experiment is indicated in the corresponding figure legends. All data are presented as mean ± SD. *p* < 0.05 was considered statistically significant. ns: not significant; ^*^ *p* < 0.05; ^**^ *p* < 0.01; ^***^ *p* < 0.001.

## Author Contributions

The author contributions according to the CRediT classification are as follows: YQZ and CH contributed to methodology. YQZ and ZBH performed the experiments. YQZ curated the data. YQZ and CH performed formal analysis. YQZ, CH, HBC, FC, and YJZ validated the study. HBC, FC, and YJZ administered the project. HBC, FC, and YJZ contributed to conceptualization. HBC, FC, and YJZ provided resources and supervised the study. YQZ and CH drafted the original manuscript. YQZ prepared the visualizations. YQZ, CH, and FC contributed to writing – review and editing. All authors read and approved the final manuscript.

## Funding

This work was supported by National Natural Science Foundation of China (82572401 and 82170199, China). Natural Science Foundation of Guangdong Province (2026A1515012256 and 2023A1515010978, China). The Guangdong Provincial Key Area R&D Program (2025B1111120001, China). Shenzhen Medical Research Fund (C2401020, China). Shenzhen Science and Technology Program (KJZD20240903101300002 and JCYJ20220818102605012, China). National Key R&D Program of China (2022YFA1104900, China).

## Ethics Statement

All animal experiments were performed in accordance with institutional guidelines and were approved by the Institutional Animal Care and Use Committee of Sun Yat‐sen University (SYSU‐IACUC‐2024‐000116).

## Conflicts of Interest

All authors declare no conflict of interest.

## Supporting information




**Supporting File 1**: advs75686‐sup‐0001‐SuppMat.docx.

**Supporting File 2**: advs75686‐sup‐0002‐FigureS1‐S3.zip.

## Data Availability

The data that support the findings of this study are available from the corresponding author upon reasonable request.

## References

[1] S. V. Rajkumar , M. A. Dimopoulos , A. Palumbo , et al., “International Myeloma Working Group Updated Criteria for the Diagnosis of Multiple Myeloma,” The Lancet Oncology 15, no. 12 (2014): e538–e548, 10.1016/s1470-2045(14)70442-5. 25439696

[2] P. Moreau , J. San Miguel , P. Sonneveld , et al., “Multiple Myeloma: ESMO Clinical Practice Guidelines for Diagnosis, Treatment and Follow‐up,” Annals of Oncology 28, no. 4 (2017): iv52–iv61, 10.1093/annonc/mdx096. 28453614

[3] E. Muchtar , A. Dispenzieri , M. A. Gertz , et al., “Treatment of AL Amyloidosis: Mayo Stratification of Myeloma and Risk‐Adapted Therapy (mSMART) Consensus Statement 2020 Update,” Mayo Clinic Proceedings 96, no. 6 (2021): 1546–1577, 10.1016/j.mayocp.2021.03.012. 34088417

[4] S. V. Rajkumar , “Multiple Myeloma: 2024 Update on Diagnosis, Risk‐Stratification, and Management,” American Journal of Hematology 99, no. 9 (2024): 1802–1824, 10.1002/ajh.27422. 38943315 PMC11404783

[5] J. T. Moore , S. M. Mettias , J. Cheung , et al., “Outcomes for Unselected, Newly Diagnosed Multiple Myeloma Patients,” Haematologica 110, no. 11 (2025): 2823, 10.3324/haematol.2025.287458. 40468965 PMC12580707

[6] R. L. Siegel , T. B. Kratzer , N. S. Wagle , H. Sung , and A. Jemal , “Cancer Statistics, 2026,” CA: A Cancer Journal for Clinicians 76, no. 1 (2026): e70043, 10.3322/caac.70043. 41528114 PMC12798275

[7] T. Yue , Y. Sun , Y. Dai , and F. Jin , “Mechanisms for Resistance to BCMA‐Targeted Immunotherapies in Multiple Myeloma,” Blood Reviews 70 (2025): 101256, 10.1016/j.blre.2025.101256. 39818472

[8] T. Hideshima , C. Mitsiades , G. Tonon , P. G. Richardson , and K. C. Anderson , “Understanding Multiple Myeloma Pathogenesis in the Bone Marrow to Identify New Therapeutic Targets,” Nature Reviews Cancer 7, no. 8 (2007): 585–598, 10.1038/nrc2189. 17646864

[9] K. Lu , W. Wang , Y. Liu , C. Xie , J. Liu , and L. Xing , “Advancements in Microenvironment‐Based Therapies: Transforming the Landscape of Multiple Myeloma Treatment,” Frontiers in Oncology 14 (2024): 1413494, 10.3389/fonc.2024.1413494. 39087026 PMC11288838

[10] K. Kasomva , K. Yadav , S. Janz , B. Dhakal , and S. Rao , “Molecular and Immunological Determinants of Long‐Term Survival in Multiple Myeloma,” Blood Advances 9, no. 20 (2025): 5134–5147, 10.1182/bloodadvances.2025016829. 40674709 PMC12550235

[11] L. Wang , M. Shi , A. Y. Sung , C. C. Yin , Y. Bai , and M. Chen , “Role of the Bone Marrow Microenvironment in Multiple Myeloma: Impact of Niches on Drug Resistance Mechanisms,” Seminars in Diagnostic Pathology 42, no. 4 (2025): 150916, 10.1016/j.semdp.2025.150916. 40440932

[12] Y. Kawano , M. Moschetta , S. Manier , et al., “Targeting the Bone Marrow Microenvironment in Multiple Myeloma,” Immunological Reviews 263, no. 1 (2015): 160–172, 10.1111/imr.12233. 25510276

[13] N. Shah , A. Chari , E. Scott , K. Mezzi , and S. Z. Usmani , “B‐Cell Maturation Antigen (BCMA) in Multiple Myeloma: Rationale for Targeting and Current Therapeutic Approaches,” Leukemia 34, no. 4 (2020): 985–1005, 10.1038/s41375-020-0734-z. 32055000 PMC7214244

[14] Y. T. Tai , C. Acharya , G. An , et al., “APRIL and BCMA Promote Human Multiple Myeloma Growth and Immunosuppression in the Bone Marrow Microenvironment,” Blood 127, no. 25 (2016): 3225–3236, 10.1182/blood-2016-01-691162. 27127303 PMC4920023

[15] E. Claudio , K. Brown , S. Park , H. Wang , and U. Siebenlist , “BAFF‐Induced NEMO‐Independent Processing of NF‐κB2 in Maturing B cells,” Nature Immunology 3, no. 10 (2002): 958–965, 10.1038/ni842. 12352969

[16] N. C. Munshi , L. D. Anderson , N. Shah , et al., “Idecabtagene Vicleucel in Relapsed and Refractory Multiple Myeloma,” New England Journal of Medicine 384, no. 8 (2021): 705–716, 10.1056/NEJMoa2024850. 33626253

[17] J. G. Berdeja , D. Madduri , S. Z. Usmani , et al., “Ciltacabtagene Autoleucel, a B‐cell Maturation Antigen‐directed Chimeric Antigen Receptor T‐cell Therapy in Patients With Relapsed or Refractory Multiple Myeloma (CARTITUDE‐1): A Phase 1b/2 Open‐label Study,” The Lancet 398, no. 10297 (2021): 314–324, 10.1016/s0140-6736(21)00933-8. 34175021

[18] P. Moreau , A. L. Garfall , N. W. C. J. van de Donk , et al., “Teclistamab in Relapsed or Refractory Multiple Myeloma,” New England Journal of Medicine 387, no. 6 (2022): 495–505, 10.1056/NEJMoa2203478. 35661166 PMC10587778

[19] H. Chen , T. Yu , L. Lin , et al., “γ‐Secretase Inhibitors Augment Efficacy of BCMA‐Targeting Bispecific Antibodies Against Multiple Myeloma Cells Without Impairing T‐cell Activation and Differentiation,” Blood Cancer Journal 12, no. 8 (2022): 118, 10.1038/s41408-022-00716-3. 35973981 PMC9381512

[20] S. A. Laurent , F. S. Hoffmann , P.‐H. Kuhn , et al., “γ‐secretase Directly Sheds the Survival Receptor BCMA From Plasma Cells,” Nature Communications 6, no. 1 (2015): 7333, 10.1038/ncomms8333. PMC4490565 26065893

[21] A. D. Cohen , A. L. Garfall , E. A. Stadtmauer , et al., “B Cell Maturation Antigen–Specific CAR T Cells are Clinically Active in Multiple Myeloma,” Journal of Clinical Investigation 129, no. 6 (2019): 2210–2221, 10.1172/jci126397. 30896447 PMC6546468

[22] C. Chung , “Role of Immunotherapy in Targeting the Bone Marrow Microenvironment in Multiple Myeloma: an Evolving Therapeutic Strategy,” Pharmacotherapy: The Journal of Human Pharmacology and Drug Therapy 37, no. 1 (2017): 129–143, 10.1002/phar.1871. 27870103

[23] N. C. Gordon , B. Pan , S. G. Hymowitz , et al., “BAFF/BLyS Receptor 3 Comprises a Minimal TNF Receptor‐Like Module That Encodes a Highly Focused Ligand‐binding Site,” Biochemistry 42, no. 20 (2003): 5977–5983, 10.1021/bi034017g. 12755599

[24] J. Moreaux , E. Legouffe , E. Jourdan , et al., “BAFF and APRIL Protect Myeloma Cells From Apoptosis Induced by Interleukin 6 Deprivation and Dexamethasone,” Blood 103, no. 8 (2004): 3148–3157, 10.1182/blood-2003-06-1984. 15070697 PMC2387243

[25] N. S. Raje , P. Moreau , E. Terpos , et al., “Phase 2 Study of Tabalumab, a Human Anti‐B‐Cell Activating Factor Antibody, With Bortezomib and Dexamethasone in Patients With Previously Treated Multiple Myeloma,” British Journal of Haematology 176, no. 5 (2017): 783–795, 10.1111/bjh.14483. 28005265

[26] C. He , M. Zhang , L. Liu , et al., “Cellular Membrane‐based Vesicles Displaying a Reconstructed B Cell Maturation Antigen for Multiple Myeloma Therapy by Dual Targeting APRIL and BAFF,” Acta Biomaterialia 143 (2022): 406–417, 10.1016/j.actbio.2022.02.028. 35218967

[27] K. Palucka and J. Banchereau , “Cancer Immunotherapy via Dendritic Cells,” Nature Reviews Cancer 12, no. 4 (2012): 265–277, 10.1038/nrc3258. 22437871 PMC3433802

[28] J. Constantino , C. Gomes , A. Falcão , M. T. Cruz , and B. M. Neves , “Antitumor Dendritic Cell–Based Vaccines: Lessons From 20 Years of Clinical Trials and Future Perspectives,” Translational Research 168 (2016): 74–95, 10.1016/j.trsl.2015.07.008. 26297944

[29] M. Ratta , F. Fagnoni , A. Curti , et al., “Dendritic Cells Are Functionally Defective in Multiple Myeloma: the Role of Interleukin‐6,” Blood 100, no. 1 (2002): 230–237, 10.1182/blood.v100.1.230. 12070032

[30] D. Gabrilovich , T. Ishida , T. Oyama , et al., “Vascular Endothelial Growth Factor Inhibits the Development of Dendritic Cells and Dramatically Affects the Differentiation of Multiple Hematopoietic Lineages in Vivo,” Blood 92, no. 11 (1998): 4150–4166. 9834220

[31] L. Zitvogel , A. Regnault , A. Lozier , et al., “Eradication of Established Murine Tumors Using a Novel Cell‐Free Vaccine: Dendritic Cell Derived Exosomes,” Nature Medicine 4, no. 5 (1998): 594–600, 10.1038/nm0598-594. 9585234

[32] J. M. Pitt , M. Charrier , S. Viaud , et al., “Dendritic Cell–Derived Exosomes as Immunotherapies in the Fight Against Cancer,” The Journal of Immunology 193, no. 3 (2014): 1006–1011, 10.4049/jimmunol.1400703. 25049431

[33] S. Viaud , C. Théry , S. Ploix , et al., “Dendritic Cell‐Derived Exosomes for Cancer Immunotherapy: What's Next?,” Cancer Research 70, no. 4 (2010): 1281–1285, 10.1158/0008-5472.Can-09-3276. 20145139

[34] E. Segura , C. Nicco , B. Lombard , et al., “ICAM‐1 on Exosomes From Mature Dendritic Cells Is Critical for Efficient Naive T‐cell Priming,” Blood 106, no. 1 (2005): 216–223, 10.1182/blood-2005-01-0220. 15790784

[35] M. B. Meads , R. A. Gatenby , and W. S. Dalton , “Environment‐mediated Drug Resistance: a Major Contributor to Minimal Residual Disease,” Nature Reviews Cancer 9, no. 9 (2009): 665–674, 10.1038/nrc2714. 19693095

[36] F. Malard , P. Neri , N. J. Bahlis , et al., “Multiple Myeloma,” Nature Reviews Disease Primers 10, no. 1 (2024): 45, 10.1038/s41572-024-00529-7. 38937492

[37] E. S. Day , T. G. Cachero , F. Qian , et al., “Selectivity of BAFF/BLyS and APRIL for Binding to the TNF Family Receptors BAFFR/BR3 and BCMA,” Biochemistry 44, no. 6 (2005): 1919–1931, 10.1021/bi048227k. 15697217

[38] M. Eslami , S. Schuepbach‐Mallepell , D. Diana , et al., “Unique and Redundant Roles of Mouse BCMA, TACI, BAFF, APRIL, and IL‐6 in Supporting Antibody‐producing Cells in Different Tissues,” Proceedings of the National Academy of Sciences 121, no. 29 (2024): 2404309121, 10.1073/pnas.2404309121. PMC11260164 38990948

[39] Y. R. Miao , K. Thakkar , C. Cenik , et al., “Developing High‐affinity Decoy Receptors to Treat Multiple Myeloma and Diffuse Large B Cell Lymphoma,” Journal of Experimental Medicine 219, no. 9 (2022): 20220214, 10.1084/jem.20220214. PMC9428257 35881112

[40] L. Di Marzo , V. Desantis , A. G. Solimando , et al., “Microenvironment Drug Resistance in Multiple Myeloma: Emerging New Players,” Oncotarget 7, no. 37 (2016): 60698–60711, 10.18632/oncotarget.10849. 27474171 PMC5312413

[41] S. Yuan , Q. Li , C. He , et al., “Anti‐BCMA–Engineered Exosomes for Bortezomib‐Targeted Delivery in Multiple Myeloma,” Blood Advances 8, no. 18 (2024): 4886–4899, 10.1182/bloodadvances.2023012464. 38875465 PMC11421322

[42] W. Fu , C. Lei , S. Liu , et al., “CAR Exosomes Derived From Effector CAR‐T Cells Have Potent Antitumour Effects and Low Toxicity,” Nature Communications 10, no. 1 (2019): 4355, 10.1038/s41467-019-12321-3. PMC6761190 31554797

[43] K. W. Witwer and J. Wolfram , “Extracellular Vesicles versus Synthetic Nanoparticles for Drug Delivery,” Nature Reviews Materials 6, no. 2 (2021): 103–106, 10.1038/s41578-020-00277-6. PMC9481198 36117545

[44] T. Redkin and V. Turubanova , “Dendritic Cell‐derived Exosomes as Anti‐cancer Cell‐free Agents: New Insights Into Enhancing Immunogenic Effects,” Frontiers in Immunology 16 (2025): 1586892, 10.3389/fimmu.2025.1586892. 40503230 PMC12151788

[45] P. Leone , S. Berardi , M. A. Frassanito , et al., “Dendritic Cells Accumulate in the Bone Marrow of Myeloma Patients Where They Protect Tumor Plasma Cells From CD8^+^ T‐cell Killing,” Blood 126, no. 12 (2015): 1443–1451, 10.1182/blood-2015-01-623975. 26185130 PMC4592278

[46] G. Bianchi and N. C. Munshi , “Pathogenesis Beyond the Cancer Clone(s) in Multiple Myeloma,” Blood 125, no. 20 (2015): 3049–3058, 10.1182/blood-2014-11-568881. 25838343 PMC4432002

[47] P. K. Myint , E. J. Park , A. Gaowa , E. Kawamoto , and M. Shimaoka , “Targeted Remodeling of Breast Cancer and Immune Cell Homing Niches by Exosomal Integrins,” Diagnostic Pathology 15, no. 1 (2020): 38, 10.1186/s13000-020-00959-3. 32305065 PMC7165434

[48] E. Verheye , J. Bravo Melgar , S. Deschoemaeker , et al., “Dendritic Cell‐Based Immunotherapy in Multiple Myeloma: Challenges, Opportunities, and Future Directions,” International Journal of Molecular Sciences 23, no. 2 (2022): 904, 10.3390/ijms23020904. 35055096 PMC8778019

[49] T. L. Whiteside , “Exosomes and Tumor‐mediated Immune Suppression,” Journal of Clinical Investigation 126, no. 4 (2016): 1216–1223, 10.1172/jci81136. 26927673 PMC4811135

[50] J. Li , J. Wang , and Z. Chen , “Emerging Role of Exosomes in Cancer Therapy: Progress and Challenges,” Molecular Cancer 24, no. 1 (2025): 13, 10.1186/s12943-024-02215-4. 39806451 PMC11727182

[51] R. Gastpar , M. Gehrmann , M. A. Bausero , et al., “Heat Shock Protein 70 Surface‐Positive Tumor Exosomes Stimulate Migratory and Cytolytic Activity of Natural Killer Cells,” Cancer Research 65, no. 12 (2005): 5238–5247, 10.1158/0008-5472.Can-04-3804. 15958569 PMC1785299

[52] M. H. Rashed , E. Bayraktar , G. K. Helal , et al., “Exosomes: from Garbage Bins to Promising Therapeutic Targets,” International Journal of Molecular Sciences 18, no. 3 (2017): 538, 10.3390/ijms18030538. 28257101 PMC5372554

[53] H. Peinado , M. Aleckovic , S. Lavotshkin , et al., “Melanoma Exosomes Educate Bone Marrow Progenitor Cells Toward a Pro‐Metastatic Phenotype Through MET,” Nature Medicine 18, no. 6 (2012): 883–891, 10.1038/nm.2753. PMC3645291 22635005

[54] F. B. Vincent , E. F. Morand , P. Schneider , and F. Mackay , “The BAFF/APRIL System in SLE Pathogenesis,” Nature Reviews Rheumatology 10, no. 6 (2014): 365–373, 10.1038/nrrheum.2014.33. 24614588

