# Supplementary material for: BCMA‐Engineered Dendritic Cell‐Derived Exosomes as Bi‐Functional Therapeutics Orchestrating Cytokine Sequestration and Immune Activation for Multiple Myeloma
Source: Adv Sci (Weinh). 2026 May 15:e75686. Online ahead of print. doi: 10.1002/advs.75686 (PMC13335986; doi:10.1002/advs.75686)
Supplement: Supplementary file 1 — Supporting File 1: advs75686‐sup‐0001‐SuppMat.docx. [file ADVS-9999-e75686-s002.docx]

**Supporting Information**

**BCMA-Engineered Dendritic Cell-Derived Exosomes as Bi-functional Therapeutics Orchestrating Cytokine Sequestration and Immune Activation for Multiple Myeloma**

*Yuqing Zeng^1 #^, Chao He^1,4 #^, Zhibin He^2 #^, Hongbo Chen^1 *^, Fang Cheng^1 *^, Yongjiang Zheng^2,3 *^*

1. School of Pharmaceutical Sciences (Shenzhen), Sun Yat-sen University, Shenzhen, 518107, China.
2. Department of Hematology, Institute of Hematology, The Third Affiliated Hospital of Sun Yat-sen University, Guangzhou, 510630, China.
3. Department of Hematology, Zhaoqing Hospital, The Third Affiliated Hospital of Sun Yat-sen University, Zhaoqing, 526000, China.
4. Department of Endocrinology, Southwest Hospital, Army Medical University (The Third Military Medical University), Chongqing 400038, China.

* Correspondence to: [zhengyj5@mail.sysu.edu.cn](mailto:zhengyj5@mail.sysu.edu.cn) (Yongjiang Zheng)

[chenhb7@mail.sysu.edu.cn](mailto:chenhb7@mail.sysu.edu.cn) (Hongbo Chen)

[chengf9@mail.sysu.edu.cn](mailto:chengf9@mail.sysu.edu.cn) (Fang Cheng) √

√ Fang Cheng will handle correspondence at all stages of refereeing and publication, also post-publication. [Tel: +86-15527709102](Tel:+86-15527709102).

*^#^* Yuqing Zeng, Chao He, and Zhibin He contributed equally to this work.

**Table S1: Primers used for RT-qPCR.**

| **Primer Name** | **Primer sequence** | |
| --- | --- | --- |
|  | **Forward (5′–3′)** | **Reverse (5′–3′)** |
| *Gapdh* | AGGTCGGTGTGAACGGATTTG | TGTAGACCATGTAGTTGAGGTCA |
| *Actb* | GCCTTCCTTCTTGGGTATGGA | ACGGATGTCAACGTCACACT |
| *Ccnb1* | TGACGTAGACGCAGATGATGG | ACTGACTGCTCTTCCTCCAGT |
| *Ccne1* | CTTATGGTGTCCTCGCTGCT | CGCACCACTGATAACCTGAGA |
| *Ccnd2* | TGAAAGAGACCATCCCGCTG | TACCAGTTCCCACTCCAGCA |
| *Bcl-2* | GTGTGGAGAGCGTCAACAGG | ATATAGTTCCACAAAGGCATCCCAG |
| *Bcl-xL* | TGCAGGTATTGGTGAGTCGG | GATCCACAAAAGTGTCCCAGC |
| *Mcl-1* | TGCCTTTGTGGCCAAACACTT | GAACTCCACAAACCCATCCCAG |
| *Cd80* | ACCCCCAACATAACTGAGTCT | TTCCAACCAAGAGAAGCGAGG |
| *Cd86* | CTGCTCATCTATACACGGTTACC | GGAAACGTCGTACAGTTCTGTG |
| *MhcII* | AGACGCCGAGTACTGGAACA | ACATTGGGCTGTTCAAGCCG |
| *Tnfrsf17 (BCMA)* | ACTGTGATCCAAGCGTGACC | GAGGCTCGTCCTTCAGGG |

**Supplementary Figures**


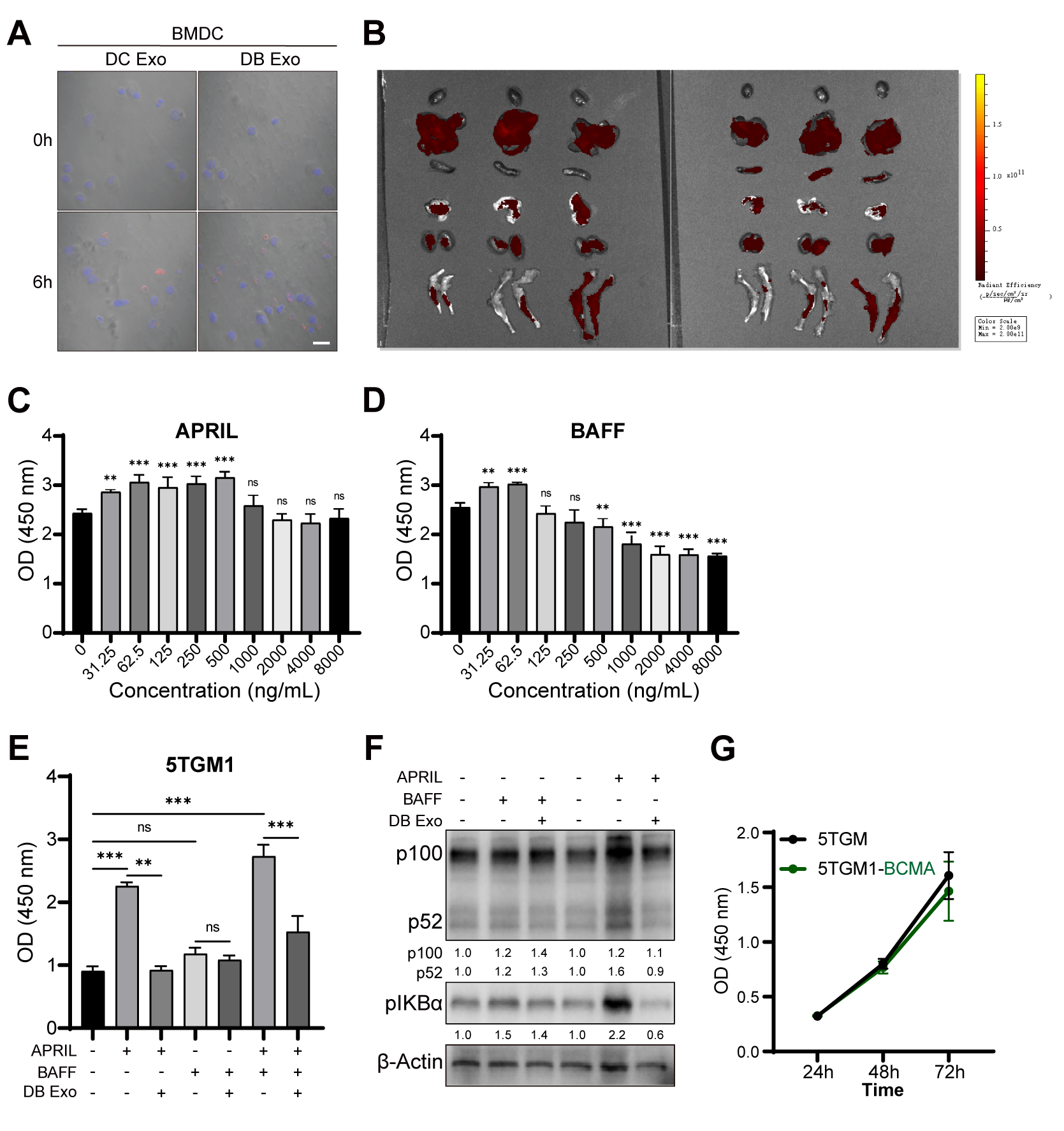


**Figure S1. (A)** CLSM images of bone marrow-derived dendritic cells (BMDCs) incubated with Cy5-labeled DC Exo or DB Exo at 0 h and 6 h. Nuclei were stained with DAPI (blue). Scale bar: 10 µm. (**B**) Representative ex vivo fluorescence images of major organs and femurs harvested 12 h after intravenous injection of Cy5-labeled exosomes are shown from two independent biodistribution experiments, corresponding to the quantitative data in **Figure 1L**. (**C**, **D**) CCK-8 assay showing concentration-dependent effects of APRIL (**C**) and BAFF (**D**) on 5TGM1 cell viability across a dose range of 31.25 to 8000 ng/mL. *n* = 3. (**E**) CCK-8 assay quantifying 5TGM1 cell viability under single-ligand (APRIL or BAFF) and combined stimulation conditions with or without DB Exo treatment at the selected proliferative concentration (50 ng/mL). *n* = 3. (**F**) Western blot analysis of non-canonical (p100 to p52 processing) and canonical (pIκBα) NF-κB signaling in 5TGM1 cells under indicated treatment conditions. Densitometric values normalized to β-Actin and expressed relative to the untreated control group (set to 1.0) are shown below each band. (**G**) Growth curves of wild-type 5TGM1 and BCMA-overexpressing 5TGM1-BCMA cells over 72 h, assessed by CCK-8 assay. *n* = 6. Dunnett's test was used for panels **C** and **D** (comparisons of each treatment concentration with the untreated control), and one-way ANOVA followed by Tukey's multiple comparisons test for panel **E**. All data are presented as mean ± SD. ***p* < 0.01, ****p* < 0.001, ns: not significant.


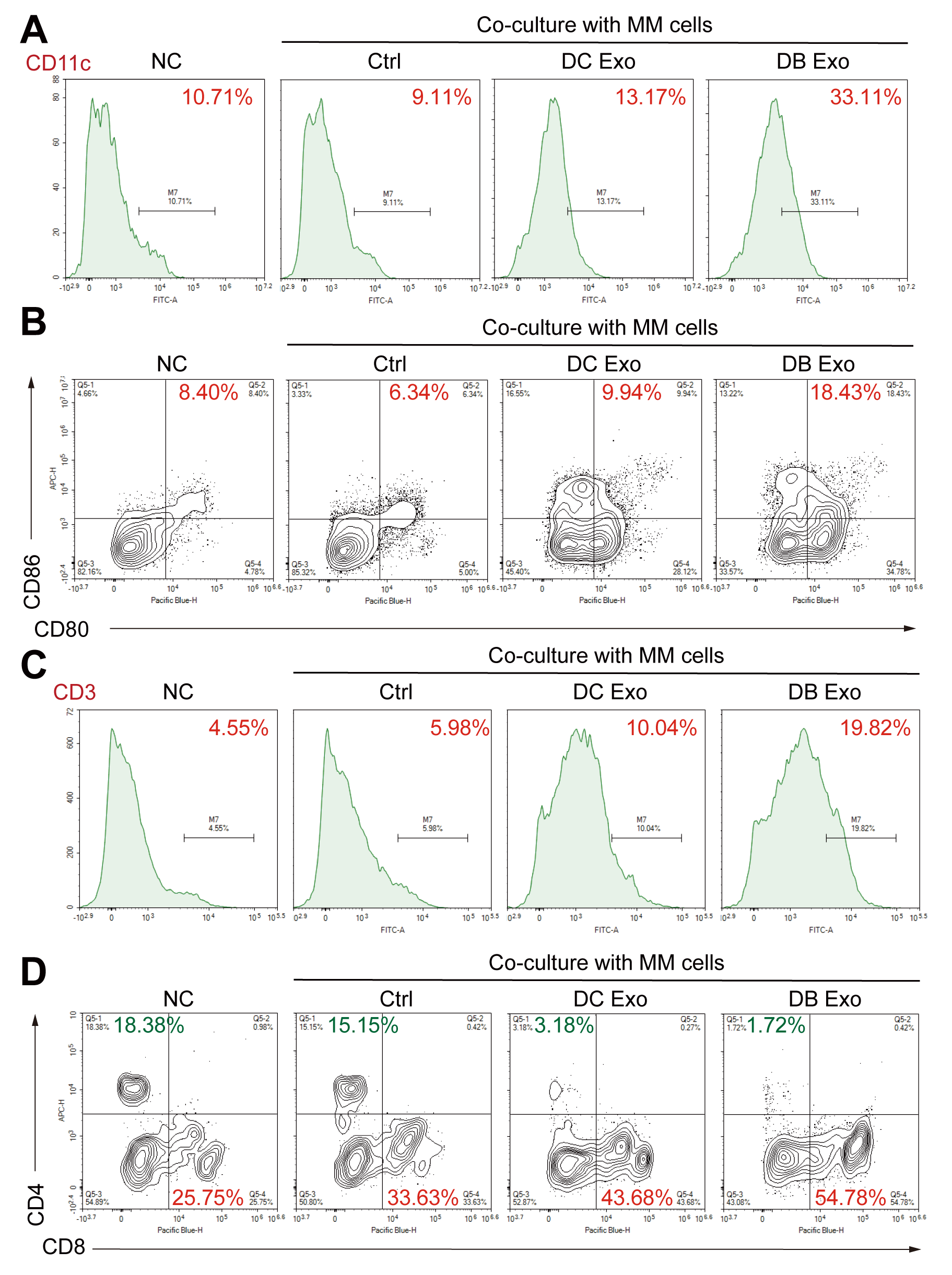


**Figure S2. Representative flow cytometry analysis for immune profiling in the bone marrow microenvironment.** (**A**) Representative flow cytometry histograms showing CD11c^+^ dendritic cells, with the percentages indicated, in the bone marrow co-culture following treatment with PBS (Ctrl), DC Exo, or DB Exo. (**B**) Representative flow cytometry plots showing CD80^+^CD86^+^ mature dendritic cells, with the proportions indicated. (**C**) Representative flow cytometry histograms showing CD3^+^ T cells, with the percentages indicated. (**D**) Representative flow cytometry plots showing CD4 and CD8 expression; the corresponding quantification of the percentage of CD8^+^ T cells is shown in **Figure 3R**. Quantitative data and statistical analyses are shown in **Figure 3**.


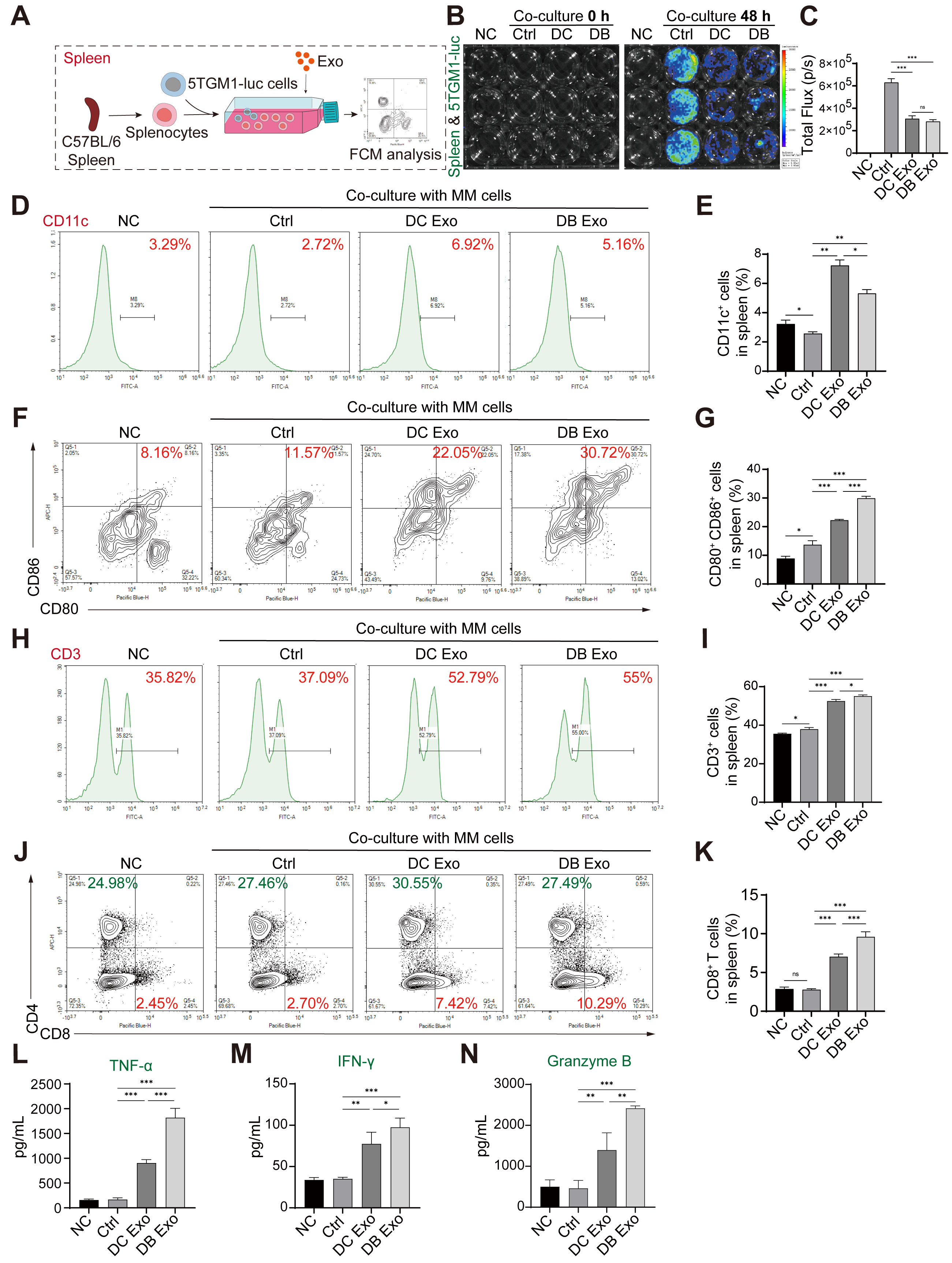


**Figure S3**. **DB Exo treatment enhances splenocyte-mediated anti-tumor immune responses ex vivo**. (**A**) Schematic diagram illustrating the ex vivo splenocyte/5TGM1-Luc co-culture system. (**B, C**) Assessment of tumor cell burden via bioluminescence imaging (**B**) and quantification of total bioluminescence flux (p/s) (**C**) after 48 h of treatment with PBS, DC Exo, or DB Exo. *n =* 3. (**D, E**) Representative flow cytometry histograms (**D**) and quantification (**E**) of the percentage of CD11c^+^ dendritic cells in splenocyte co-culture. *n =* 3. (**F, G**) Representative flow cytometry plots (**F**) and quantification (**G**) of the proportion of CD80^+^CD86^+^ mature dendritic cells. *n =* 3. (**H, I**) Representative flow cytometry histograms (**H**) and quantification (**I**) of the percentage of CD3^+^ T cells. *n =* 3. (**J, K**) Representative flow cytometry plots (**J**) and quantification of the percentage of CD8^+^ T cells (**K**). *n =* 3. (**L-N**) ELISA quantification of TNF-α (**L**), IFN-γ (**M**), and Granzyme B (**N**) concentrations in the co-culture supernatants. *n =* 3. Statistical significance was analyzed using one-way ANOVA followed by Tukey’s multiple comparisons test for panels **C**, **E**, **G**, **I**, **K**, **L**, **M**, and **N**. All data are presented as mean ± SD. **p* < 0.05, ***p* < 0.01, ****p* < 0.001, ns: not significant.
