# Supplementary figures and images for: BCMA‐Engineered Dendritic Cell‐Derived Exosomes as Bi‐Functional Therapeutics Orchestrating Cytokine Sequestration and Immune Activation for Multiple Myeloma
Source: Adv Sci (Weinh). 2026 May 15:e75686. Online ahead of print. doi: 10.1002/advs.75686 (PMC13335986; doi:10.1002/advs.75686)

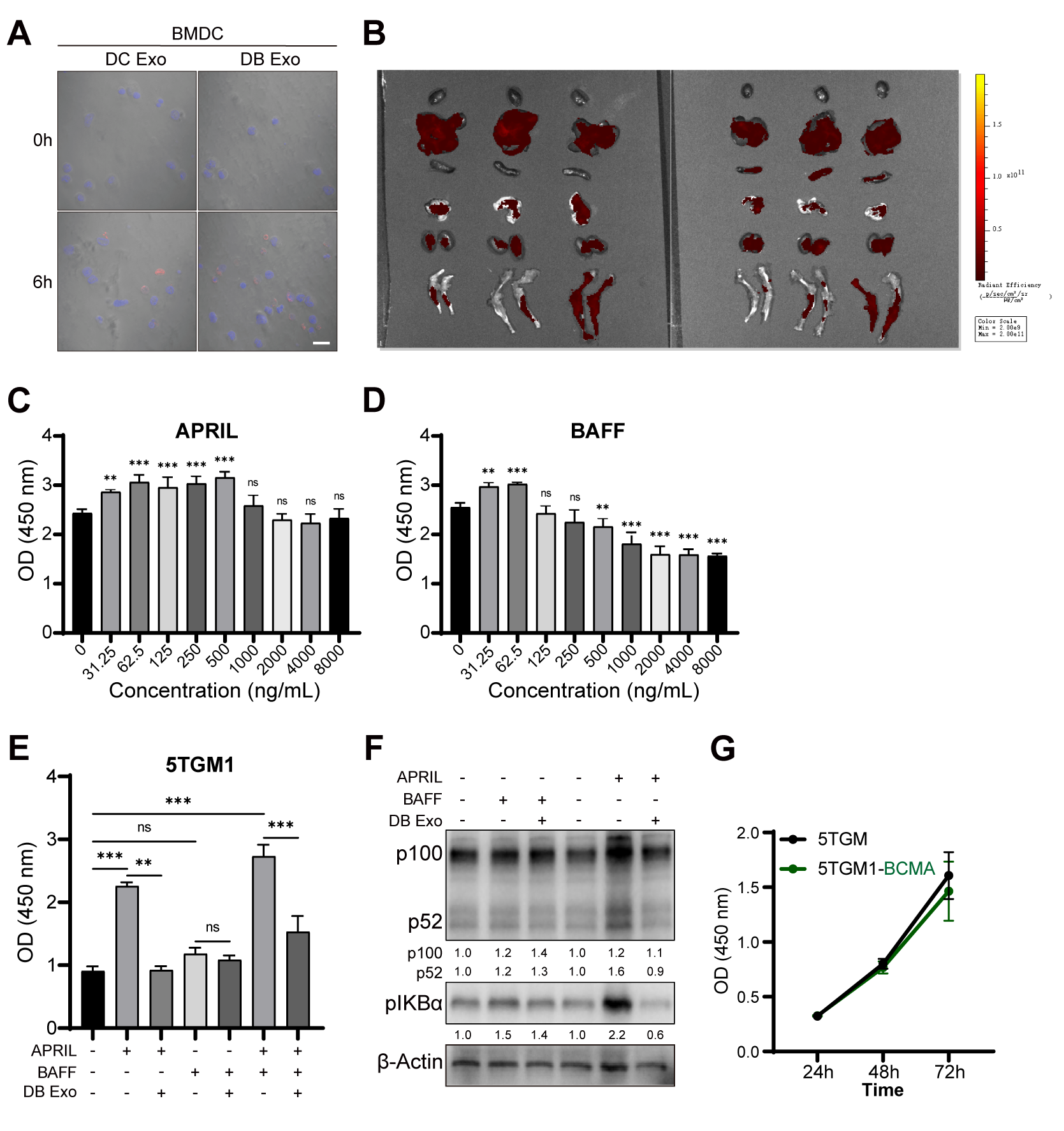

Supplement: Supplementary file 2 — Supporting File 2: advs75686‐sup‐0002‐FigureS1‐S3.zip. [file ADVS-9999-e75686-s001.zip › Figure-S1.tif]

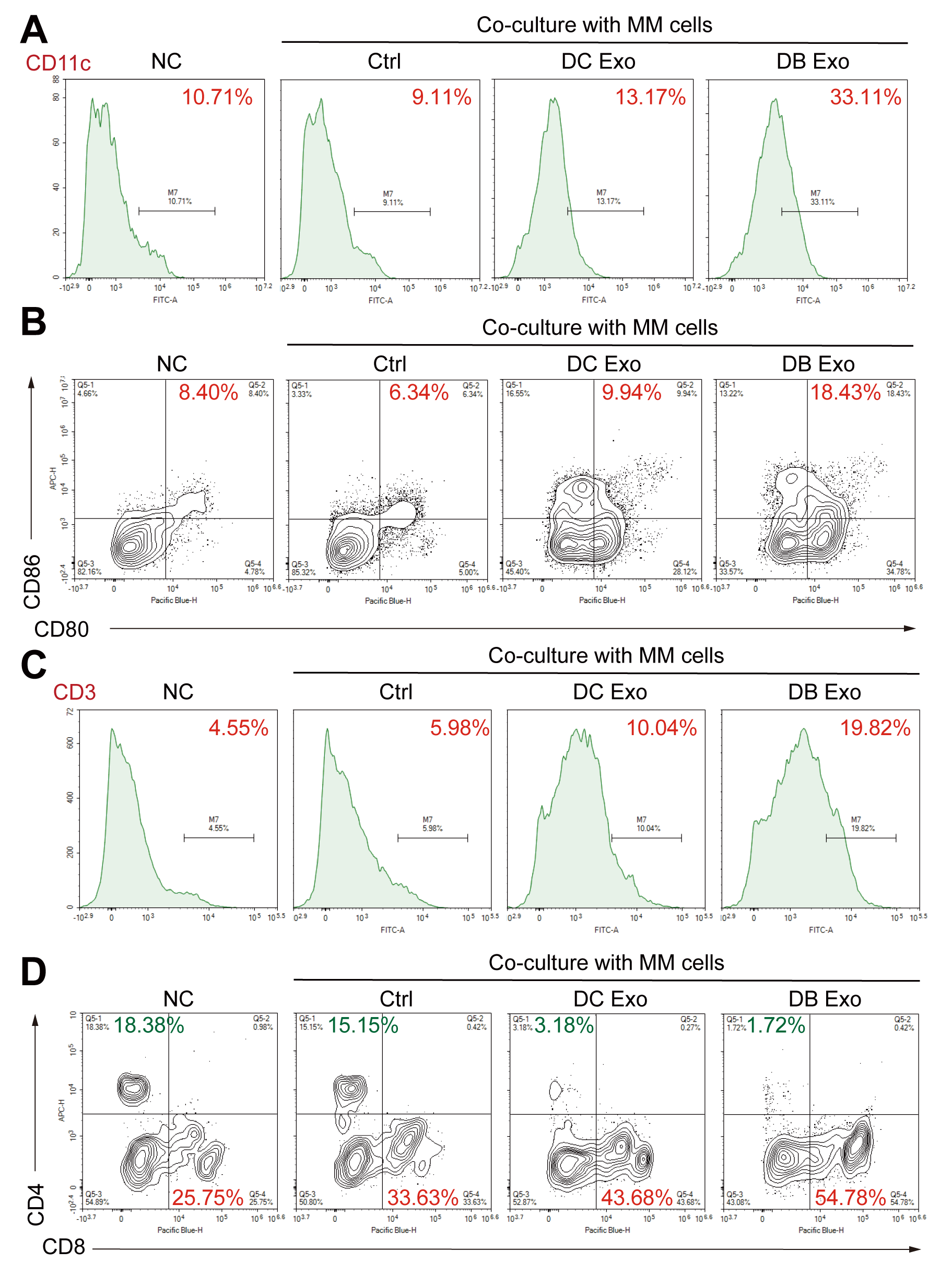

Supplement: Supplementary file 2 — Supporting File 2: advs75686‐sup‐0002‐FigureS1‐S3.zip. [file ADVS-9999-e75686-s001.zip › Figure-S2.tif]

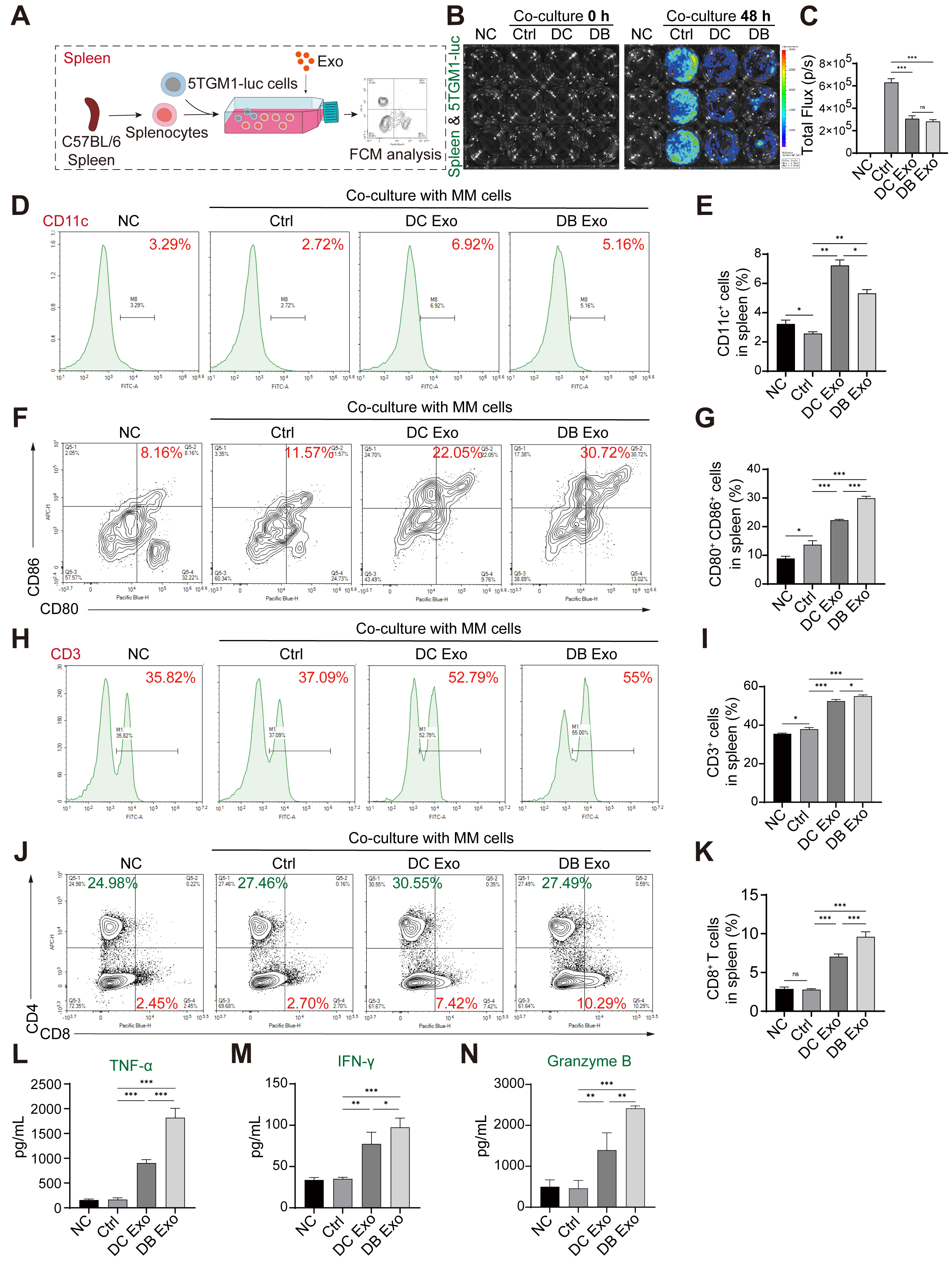

Supplement: Supplementary file 2 — Supporting File 2: advs75686‐sup‐0002‐FigureS1‐S3.zip. [file ADVS-9999-e75686-s001.zip › Figure-S3.tif]
